# Genome-wide Study Identifies Association between HLA-B^∗^55:01 and Self-Reported Penicillin Allergy

**DOI:** 10.1016/j.ajhg.2020.08.008

**Published:** 2020-09-03

**Authors:** Kristi Krebs, Jonas Bovijn, Neil Zheng, Maarja Lepamets, Jenny C. Censin, Tuuli Jürgenson, Dage Särg, Erik Abner, Triin Laisk, Yang Luo, Line Skotte, Frank Geller, Bjarke Feenstra, Wei Wang, Adam Auton, 23andMe Research Team, Michelle Agee, Stella Aslibekyan, Robert K. Bell, Katarzyna Bryc, Sarah K. Clark, Sarah L. Elson, Kipper Fletez-Brant, Pierre Fontanillas, Nicholas A. Furlotte, Pooja M. Gandhi, Karl Heilbron, Barry Hicks, David A. Hinds, Karen E. Huber, Ethan M. Jewett, Yunxuan Jiang, Aaron Kleinman, Keng-Han Lin, Nadia K. Litterman, Marie K. Luff, Jennifer C. McCreight, Matthew H. McIntyre, Kimberly F. McManus, Joanna L. Mountain, Sahar V. Mozaffari, Priyanka Nandakumar, Elizabeth S. Noblin, Carrie A.M. Northover, Jared O’Connell, Aaron A. Petrakovitz, Steven J. Pitts, G. David Poznik, J. Fah Sathirapongsasuti, Anjali J. Shastri, Janie F. Shelton, Suyash Shringarpure, Chao Tian, Joyce Y. Tung, Robert J. Tunney, Vladimir Vacic, Xin Wang, Amir S. Zare, Soumya Raychaudhuri, Tõnu Esko, Andres Metspalu, Sven Laur, Dan M. Roden, Wei-Qi Wei, Michael V. Holmes, Cecilia M. Lindgren, Elizabeth J. Phillips, Reedik Mägi, Lili Milani, João Fadista

**Affiliations:** 1 Estonian Genome Center, Institute of Genomics, University of Tartu, Tartu 51010, Estonia; 2 Institute of Molecular and Cell Biology, University of Tartu, Tartu 51010, Estonia; 3 Wellcome Centre for Human Genetics, Nuffield Department of Medicine, University of Oxford, Oxford OX3 7BN, UK; 4 Big Data Institute at the Li Ka Shing Centre for Health Information and Discovery, University of Oxford, Oxford OX3 7FZ, UK; 5 Department of Biomedical Informatics, Vanderbilt University Medical Center, Nashville, TN 37232, USA; 6 Institute of Computer Science, University of Tartu, Tartu 51009, Estonia; 7 Division of Rheumatology, Inflammation and Immunity, Brigham and Women’s Hospital, Harvard Medical School, Boston, MA 02115, USA; 8 Division of Genetics, Brigham and Women’s Hospital, Harvard Medical School, Boston, MA 02115, USA; 9 Broad Institute of MIT and Harvard, Cambridge, MA 02142, USA; 10 Department of Biomedical Informatics, Harvard Medical School, Boston, MA 02115, USA; 11 Center for Data Sciences, Brigham and Women’s Hospital, Harvard Medical School, Boston, MA 02115, USA; 12 Department of Epidemiology Research, Statens Serum Institut, Copenhagen 2300, Denmark; 13 23andMe, Inc., Sunnyvale, CA 94086, USA; 14 Centre for Genetics and Genomics Versus Arthritis, Manchester Academic Health Science Centre, University of Manchester, Manchester M13 9PT, UK; 15 STACC, Tartu 51009, Estonia; 16 Department of Medicine, Vanderbilt University Medical Center, Nashville, TN 37232, USA; 17 National Institute for Health Research Oxford Biomedical Research Centre, Oxford University Hospitals NHS Foundation Trust, John Radcliffe Hospital, Oxford OX3 7LE, UK; 18 Clinical Trial Service Unit and Epidemiological Studies Unit (CTSU), Nuffield Department of Population Health, University of Oxford, Oxford OX3 7LF, UK; 19 Medical Research Council Population Health Research Unit (MRC PHRU), Nuffield Department of Population Health, University of Oxford, Oxford OX3 7LF, UK; 20 Program in Medical and Population Genetics, Broad Institute, Cambridge, MA 02142, USA; 21 Department of Pharmacology, Vanderbilt University School of Medicine, TN 37232, USA; 22 Institute for Immunology & Infectious Diseases, Murdoch University, Murdoch, WA 6150, Australia; 23 Department of Clinical Sciences, Lund University Diabetes Centre, 214 28 Malmö, Sweden; 24 Institute for Molecular Medicine Finland (FIMM), University of Helsinki, Helsinki 00014, Finland

**Keywords:** GWAS, penicillin allergy, HLA-B∗55:01, PTPN22, EHR, pharmacogenomics, UKBB, EstBB, BioVu, 23andMe

## Abstract

Hypersensitivity reactions to drugs are often unpredictable and can be life threatening, underscoring a need for understanding their underlying mechanisms and risk factors. The extent to which germline genetic variation influences the risk of commonly reported drug allergies such as penicillin allergy remains largely unknown. We extracted data from the electronic health records of more than 600,000 participants from the UK, Estonian, and Vanderbilt University Medical Center’s BioVU biobanks to study the role of genetic variation in the occurrence of self-reported penicillin hypersensitivity reactions. We used imputed SNP to HLA typing data from these cohorts to further fine map the human leukocyte antigen (HLA) association and replicated our results in 23andMe’s research cohort involving a total of 1.12 million individuals. Genome-wide meta-analysis of penicillin allergy revealed two loci, including one located in the HLA region on chromosome 6. This signal was further fine-mapped to the HLA-B^∗^55:01 allele (OR 1.41 95% CI 1.33–1.49, p value 2.04 × 10^−31^) and confirmed by independent replication in 23andMe’s research cohort (OR 1.30 95% CI 1.25–1.34, p value 1.00 × 10^−47^). The lead SNP was also associated with lower lymphocyte counts and *in silico* follow-up suggests a potential effect on T-lymphocytes at HLA-B^∗^55:01. We also observed a significant hit in *PTPN22* and the GWAS results correlated with the genetics of rheumatoid arthritis and psoriasis. We present robust evidence for the role of an allele of the major histocompatibility complex (MHC) I gene *HLA-B* in the occurrence of penicillin allergy.

## Introduction

Adverse drug reactions (ADRs) are common in clinical practice and are associated with high morbidity and mortality. A meta-analysis of prospective studies in the US revealed the incidence of serious ADRs to be 6.7% among hospitalized patients and the cause of more than 100,000 deaths annually.^1^ In Europe, ADRs are responsible for 3.5% of all hospital admissions, with 10.1% of patients experiencing ADRs during hospitalization and 197,000 fatal cases per year.^2^^,^^3^ In the US, the cost of a single ADR event falls between 1,439 to 13,462 USD.^4^

ADRs are typically divided into two types of reactions. Type A reactions are more predictable and related to the pharmacological action of a drug, whereas type B reactions are idiosyncratic, less predictable, largely dose independent, and typically driven by hypersensitivity reactions involving the immune system.^5^ Although type B reactions are less frequent (<20%) than type A reactions, they tend to be more severe and more often lead to the withdrawal of a drug from the market.^6^ One of the most common causes of type B reactions are antibiotics,^5^ typically from the beta-lactam class, with the prevalence of penicillin allergy estimated to be as high as 25% in some settings.^7^^,^^8^ Despite the relative frequency of such reactions, there are very few studies of the genetic determinants of penicillin allergy.^9^^,^^10^ This underscores the need for a better understanding of the mechanisms and risk factors, including the role of genetic variation, that contribute to these reactions.

The increasing availability of genetic and phenotypic data in large biobanks provides an opportune means for investigating the role of genetic variation in drug-induced hypersensitivity reactions. In the present study, we sought to identify genetic risk factors underlying penicillin-induced hypersensitivity reactions by harnessing data from the Estonian Biobank (EstBB), UK Biobank (UKBB), and Vanderbilt University Medical Center’s (VUMC) DNA Biobank (BioVU), with further replication in the 23andMe research cohort.

## Subjects and Methods

### Study Subjects and Phenotype Definitions

We studied individual-level genotypic and phenotypic data of 52,000 participants from the Estonian Biobank (EstBB), 500,000 participants from UK Biobank (UKBB), and a subset of 67,323 individuals from BioVU, the VUMC biorepository linked to de-identified electronic health records with self-reported European ancestry.^11^ EstBB, UKBB, and BioVU are population- or hospital-based cohorts, providing a rich variety of phenotypic and health-related information collected for each participant. All participants have signed a consent form to allow follow-up linkage. In UKBB and EstBB we extracted information on penicillin allergy by searching the records of the participants for the Z88.0 ICD10 code indicating patient-reported allergy status to penicillin. Information on phenotypic features like age and gender were obtained from the biobank recruitment records. We also extracted likely penicillin allergies in EstBB from the recruitment questionnaires and free text fields of the electronic health records (EHRs) using a rule-based approach (see Supplemental Subjects and Methods for further details). In BioVU there were no records of Z88.0 diagnoses, so we used drug allergy labels from the allergy section of the EHRs, which includes adverse drug reactions reported by an individual or observed by the health care provider (Supplemental Subjects and Methods).

This study was approved by the Research Ethics Committee of the University of Tartu (Approval number 288/M-18) and conducted using the UK Biobank Resource under Application Number 11867.

### Genome-wide Study and Meta-analysis

The details on genotyping, quality control, and imputation are fully described elsewhere for EstBB^12^^,^^13^ and UKBB;^14^ see Supplemental Subjects and Methods for further details. In EstBB, we conducted the penicillin GWAS on 44,348 individuals, including 1,320 case subjects with self-reported allergy to beta-lactam drugs or penicillin and 43,028 control subjects. In the UKBB, GWAS on penicillin allergy (defined using ICD-10 code Z88.0) was performed among 15,782 case subjects and 370,782 control subjects. In BioVU, GWAS on penicillin allergy (defined using drug allergy labels in the EHR) was performed among 12,294 case subjects and 38,284 control subjects. For all three cohorts, the GWAS was performed with SAIGE^15^ including related individuals and adjusting for the first ten principal components (PCs) of the genotype matrix, as well as for age or birth year, sex (see Supplemental Subjects and Methods), and in BioVU, additionally for EHR length (years). We performed meta-analysis of 19,724,685 markers (with minor allele frequency [MAF] > 0.1%) and SNP effect estimates and their standard errors were combined in a fixed effects model with the inverse variance weighted method using the METAL software.^16^ Results were visualized with the R software (3.3.2) (see Web Resources).

### HLA-Typing

HLA imputation of the EstBB genotype data was performed at the Broad Institute using the SNP2HLA tool.^17^ The imputation was done for genotype data generated on the Global Screening Array v1, and after quality control the four-digit HLA alleles of 22,554 individuals were used for analysis. In UKBB we used four-digit imputed HLA data released by UKBB (see Web Resources).^14^ The imputation process, performed using HLA^∗^IMP:02,^18^ is described fully elsewhere^14^ and in the Supplemental Subjects and Methods. For the BioVU cohort, four-digit HLA-typing was imputed from SNP data with the SNP2HLA tool (Supplemental Subjects and Methods).

We performed separate additive logistic regression analysis with the called HLA alleles using R *glm* function in EstBB, UKBB, and BioVU (see Supplemental Subjects and Methods for further details). Meta-analysis of 164 HLA alleles present in all three cohorts was performed with the GWAMA software tool.^19^ A Bonferroni-corrected p value threshold of 3.05 × 10^−4^ was applied based on the number of tested alleles (0.05/164).

For detection of the strongest tagging SNP for the HLA-B^∗^55:01 allele, we calculated Pearson correlation coefficients between the HLA-B^∗^55:01 allele and all the SNPs within ±50 kb of the *HLA-B* region using the *cor* function in R (3.3.2) (see Web Resources).

### HLA-B^∗^55:01 Replication

We performed replication analysis of the HLA-B^∗^55:01 allele in 87,996 case subjects and 1,031,087 control subjects of European ancestry (close relatives removed) from the 23andMe research cohort using an additive logistic regression model (see details in the Supplemental Subjects and Methods). The self-reported phenotype of penicillin allergy was defined based on questionnaire data as a positive allergy test or allergic symptoms related to penicillin exposure (see Supplemental Subjects and Methods for further details). Meta-analysis of the HLA-B^∗^55:01 association across the four cohorts was performed with the GWAMA software tool^19^ and results were visualized with R software (3.3.2) (see Web Resources).

## Results

### Genome-wide Association Analysis of Penicillin Allergy

To discover genetic factors that may predispose to penicillin allergy, we conducted a genome-wide association study (GWAS) of 19.7 million single-nucleotide polymorphisms (SNPs) and insertions/deletions in UKBB, EstBB, and BioVu (MAF in all cohorts > 0.1%) among individuals with European ancestry. Case subjects were defined as participants with a Z88.0 ICD10 code (“Allergy status to penicillin”), which indicates a reported history of penicillin allergy (previously ICD9 “personal history of allergy to penicillin”). In total, we identified 15,782 individuals (4.1% of the total cohort size of 386,564) in UKBB with this diagnostic code. However, the corresponding number of case subjects in EstBB was only 7 (0.01% of the total cohort size of 51,936) and zero in BioVu, suggesting heterogeneity in the use of the Z88.0 ICD10 code in different countries. We therefore also identified participants that had reported drug allergy at recruitment in EstBB and categorized the EstBB self-reported reactions by drug class, using the Anatomical Therapeutic Chemical (ATC) Classification System code J01C^∗^ (beta-lactam antibacterials, penicillins) to match this to the respective Z88.0 ICD10 code. We also extracted 321 individuals with mentions of penicillin allergy in the free text fields of their EHR. This resulted in 1,320 (2.5%) case subjects with penicillin allergy in EstBB. We validated the approach in EstBB by evaluating the association between the number of filled (i.e., prescribed and purchased) penicillin (using the ATC code J01C^∗^) prescriptions per person and self-reported penicillin allergy. Using Poisson regression analysis, we identified a negative association among individuals with self-reported allergy in EstBB on the number of filled penicillin prescriptions (p value 2.41 × 10^−15^, estimate −0.18, i.e., 16% lower penicillin prescription count for individuals with penicillin allergy). In BioVU, we used drug allergy labels from the allergy section of the EHR to identify 12,294 case subjects (18.3% of the total cohort of genotyped individuals of 67,323), which is consistent with previous penicillin allergy reports using drug allergy labels.^20^ To characterize the proportion of severe reactions (anaphylaxis) to penicillin among our phenotype, we analyzed the self-reported reactions among 1,017 individuals in EstBB and found that around 3% (n = 31) of the participants reported anaphylaxis and 4% (n = 42) some form of breathing difficulties. In the BioVU cohort, 5% of participants (n = 673 out of 12,294 with penicillin allergy label) reported anaphylaxis. These figures indicate that our phenotype likely captures less-severe forms of penicillin hypersensitivity.

**Figure 1:**
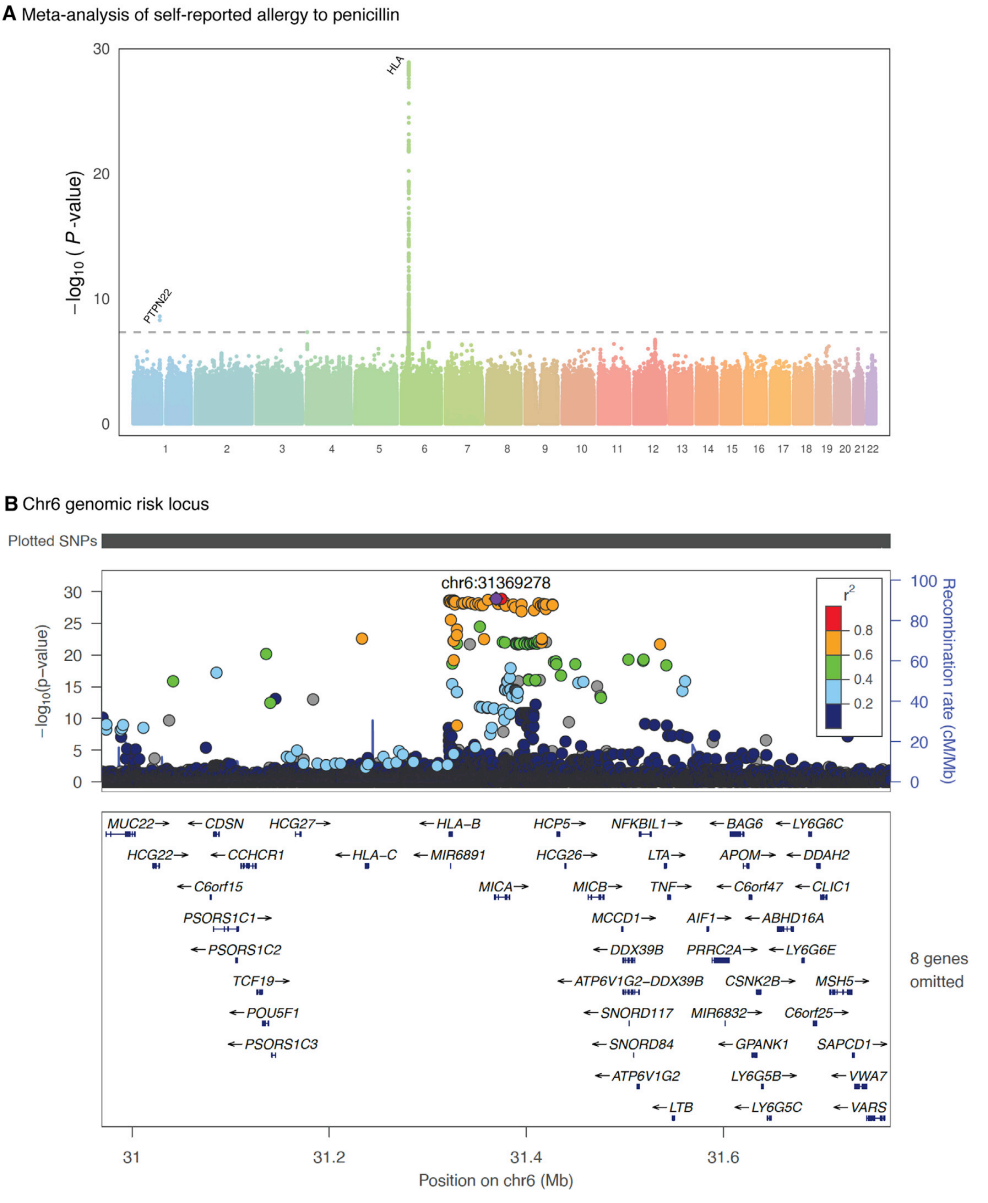
Manhattan Plot and HLA Locus of the Genome-wide Association Study of Penicillin Allergy The X axes indicate chromosomal positions and Y axes −log_10_ of the p Values. (A) Each dot represents a single-nucleotide polymorphism (SNP). The dotted line indicates the genome-wide significance (p value < 5.0 × 10^−8^) p value threshold. (B) SNPs are colored according to their linkage disequilibrium (LD; based on the 1000 Genomes phase3 EUR reference panel) with the lead SNP. The SNP marked with a purple diamond is the lead SNP rs114892859.

We then meta-analyzed the results of the GWASes in these three cohorts and identified two genome-wide significant (p < 5 × 10^−8^) signals for penicillin allergy. The top hit on chromosome 6 was located in the major histocompatibility complex (MHC) region (rs114892859, MAF(EstBB) = 0.7%, MAF(UKBB) = 2%, MAF(BioVU) = 2%; p value 1.29 × 10^−29^; OR 1.47 95% CI 1.38–1.57) (Figures 1A and S1, Table S1). We also identified a further signal for rs2476601, a missense variant in *PTPN22* on chromosome 1 (p value 2.68 × 10^−9^; OR 1.09 95% CI 1.06–1.12).

### Fine-Mapping the Penicillin Allergy-Associated HLA Locus

To further characterize the identified association with penicillin allergy, we performed a functional annotation analysis with FUMA (Functional Mapping and Annotation of Genome-Wide Association Studies).^21^ We detected an independent intronic lead SNP for the penicillin allergy meta-analysis (GWAS lead variant rs114892859, p value 1.29 × 10^−29^) in *MICA* (Figure 1B). When testing the SNP for expression quantitative trait locus (eQTL) associations in blood based on data from the eQTLGen Consortium,^22^ the variant appeared to be associated with the expression levels of several nearby genes, with the most significant being *PSORS1C3* (p value 8.10 × 10^−62^) and *MICA* (p value 1.21 × 10^−52^) (Table S2). We further performed an *in silico* investigation of the lead SNP rs114892859 and its best proxy (rs144626001, the only proxy with r^2^ > 0.9 in UKBB and EstBB) in HaploReg v.4 to explore annotations and impact of the non-coding variant.^23^ rs114892859 in particular had several annotations indicative of a regulatory function, including its location in both promoter and enhancer marks in T cells and evidence of RNA polymerase II binding.^24^^,^^25^ Interestingly, its proxy is more likely to be deleterious based on the scaled Combined Annotation Dependent Depletion (CADD) score (scaled score of 15.78 for rs144626001 (C/T) and 4.47 for rs114892859 (G/T)).^26^^,^^27^ To assess the association of the rs114892859 variant with self-reported penicillin allergy in non-European ancestries, we used the recently developed Pan-UKB resource (see Web Resources) and retrieved summary statistics for individuals of Central/South Asian, African, East Asian, and Middle Eastern (Table S3) ancestries. We did not find an association with penicillin allergy in these other ancestry groups. Neither did we find any association of the rs114892859 variant with penicillin allergy (p value 0.288; OR 0.67 95% CI 0.14–1.19) in a subset of 14,416 BioVU individuals with self-reported African ancestry, including 1,894 case subjects and 9,539 control subjects. Nevertheless, these sample sizes are substantially smaller than the European-ancestry groups we studied and larger cohorts of diverse ancestries will be needed to provide more definitive insights.

Due to the high LD in the MHC region, we used imputed SNP to HLA typing data available at four-digit resolution^28^ for up to 22,554, 488,377, and 67,323 individuals from the Estonian, UK, and BioVU cohorts, respectively, to further fine-map the identified HLA association with penicillin allergy. In all cohorts a shared total of 104 alleles at four-digit level were present for all of the MHC class I genes (*HLA-A, HLA-B, HLA-C*) and 60 alleles for three of the classical MHC class II genes (*HLA-DRB1, HLA-DQA1, HLA-DQB1*). To assess the variation in the frequencies of the HLA alleles in different populations, we compared the obtained allele frequencies in EstBB and UKBB (Table S4) with the frequencies of HLA alleles in different European, Asian, and African populations reported in the HLA frequency database (Figures S2 and S3, Table S5).

We then used an additive logistic regression model to test for associations between different four-digit HLA alleles and penicillin allergy in UKBB, EstBB, and BioVU. The results from these three cohorts were meta-analyzed, using a Bonferroni-corrected p value threshold (0.05/164 = 3.05 × 10^−4^, where 164 is the number of meta-analyzed HLA alleles). One of the two results that surpassed the threshold had discordant effects in the tested cohorts (Table S6). The only association with the same directional effect in all three cohorts that we detected for penicillin allergy was the HLA-B^∗^55:01 allele (p value 2.04 × 10^−31^; OR 1.41 95% CI 1.33–1.49; Table S6), which is tagged (r^2^ > 0.95) by the GWAS lead variant rs114892859 (Table S7). We performed a separate meta-analysis for the HLA-B^∗^55:01 allele in all case subjects from BioVU and EstBB (p value 1.98 × 10^−8^; OR 1.32 95% CI 1.20–1.45) and compared it to a meta-analysis where severe reactions of anaphylaxis were excluded. Despite the smaller sample size, the estimates from this analysis were similar (p value 1.28 × 10^−8^; OR 1.33 95% CI 1.20–1.46), indicating that the association is not driven by more severe hypersensitivity reactions.

### Replication of the HLA-B^∗^55:01 Association with Penicillin Allergy

**Figure 2:**
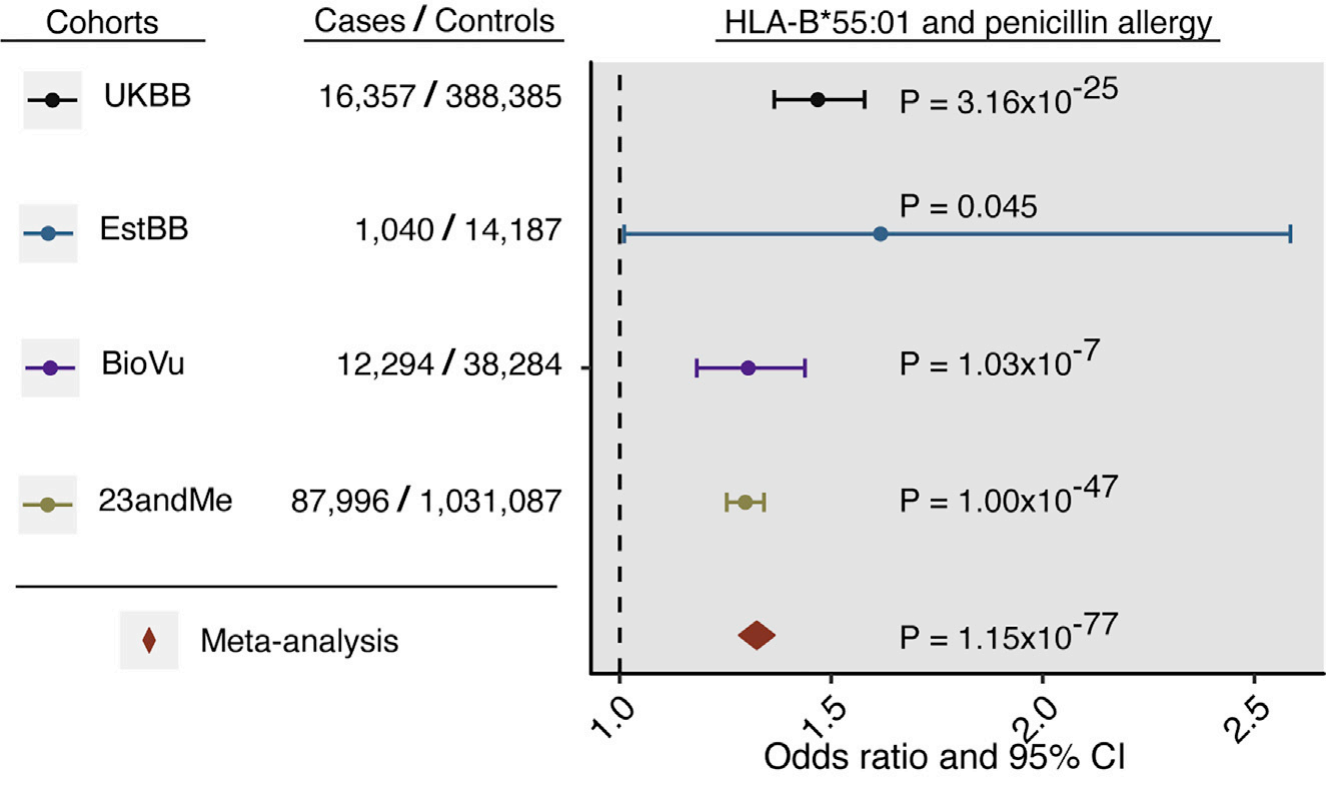
HLA-B^∗^55:01 Allele Association with Self-Reported Penicillin Allergy The odds ratios (dots) and 95% confidence intervals (CI, horizontal lines) for the association of the HLA allele with penicillin allergy are presented. The plot is annotated with p values and case-control numbers. Color coding indicates the results for discovery cohorts UKBB (black), EstBB (blue), and BioVU (purple) and replication results of the HLA-B^∗^55:01 allele in the 23andMe research cohort (green). Results of the meta-analysis of all four cohorts is indicated with a diamond (red). Self-reported penicillin allergy is defined as ICD10 code Z88.0 (UKBB), reported drug allergy labels from the allergy section of the EHR (BioVU), reported allergy to drugs in ATC J01C^∗^ class (EstBB), or reported allergy to penicillin (23andMe).

To further confirm association with penicillin allergy, we analyzed the association of the HLA-B^∗^55:01 allele with self-reported penicillin allergy among 87,996 case subjects and 1,031,087 control subjects of European ancestry from the 23andMe research cohort. We observed an association (p value 1.00 × 10^−47^; OR 1.30 95% CI 1.25–1.34; Figure 2) with a similar effect size as seen for the HLA-B^∗^55:01 allele in the meta-analysis of the EstBB, UKBB, and BioVU. Meta-analysis of estimates for HLA-B^∗^55:01 from the discovery and replication cohorts demonstrated a 33% higher relative odds of penicillin allergy among carriers of the allele (p value 1.15 × 10^−77^; OR 1.33 95% CI 1.29–1.37; Figure 2).

### Further Associations at HLA-B^∗^55:01

Finally, we used the Open Targets Genetics platform’s UKBB PheWAS data^29^ to further characterize the association of the GWAS lead variant (and HLA-B^∗^55:01 allele tag-SNP) rs114892859 with other traits. We found associations with lower lymphocyte counts (p value 9.21 × 10^−14^, −0.098 cells per nanoliter, per allergy-increasing T allele) and lower white blood cell counts (p value 3.17 × 10^−9^, −0.078 cells per nanoliter, per allergy-increasing T allele). To confirm this finding, we extracted data on lymphocyte counts from the EHR data of 4,567 EstBB participants (see Supplemental Subjects and Methods) and observed the same inverse association of the HLA-B^∗^55:01 allele with lymphocyte counts (−0.148 cells per nanoliter, per T allele; p value = 0.047).

To investigate the possible functional impact of the HLA-B^∗^55:01 allele, we compared the amino acid sequence of all the *HLA-B* alleles commonly present in Estonian and UK biobank (see Supplemental Subjects and Methods). Only one allele that was represented in our populations, HLA-B^∗^56:01, shared a high sequence similarity (>99%) with HLA-B^∗^55:01, while all the other 46 alleles shared 86.5%–93% of the amino acids within the antigen-binding cleft (Table S8). Further analysis revealed that HLA-B^∗^56:01 and HLA-B^∗^55:01 differ by only two amino acids in the α2 domain: p.Glu152Val and p.Thr163Leu (Figure S4). We did not observe an association between the HLA-B^∗^56:01 allele and penicillin allergy (p value = 0.24), which might suggest that the two amino acid differences may have functional relevance in penicillin allergy. However, despite the large number of case subjects in our study, power was limited to rule out an association with HLA-B^∗^56:01, as it is only present at a frequency of 0.3% in European populations.

To get closer to the possible endophenotypes tagging the identified associations, we investigated genetic correlation of self-reported penicillin allergy with studies on autoimmune and hematological traits using LDhub (Table S9).^30^ The analysis pointed toward genetic correlation (r_g_) of 0.35 (p value 3.65 × 10^−7^) between self-reported penicillin allergy and rheumatoid arthritis (RA). This result was virtually unchanged when we excluded 1 Mb around *PTPN22* (r_g_ = 0.35, p value 6.13 × 10^−6^), a known RA risk locus. Since we detected this genetic correlation between RA and self-reported penicillin allergy, we redid the penicillin allergy association analysis for the HLA-B^∗^55:01 allele among only RA case subjects in UKBB (468 penicillin allergy case subjects and 4,065 control subjects). The effect estimate for the HLA-B^∗^55:01 allele was similar to that from the whole UKBB cohort (p value 0.032; OR 1.57 95% CI 1.04–2.38). Because LDhub did not have data for psoriasis, we further used summary statistics of the GWAS meta-analysis available for psoriasis from the PAN UKBB resource (2,868 case subjects and 417,663 control subject subjects). The genetic correlation of the GWAS meta-analysis of penicillin allergy with psoriasis was 0.44 (p value 0.002). In summary, our results suggest that the self-reported penicillin allergy phenotype could be tagging a less severe, T cell-mediated, delayed-type penicillin allergy, and that it may involve an autoimmune component.

## Discussion

In the present study, we identify associations of the HLA-B^∗^55:01 allele and a missense variant rs2476601 in *PTPN22* with self-reported penicillin allergy using data from four large cohorts: UKBB, EstBB, BioVu, and 23andMe. Hypersensitivity or allergic reactions to medications are type B adverse drug reactions that are known to be mediated by the immune system. One major driver of hypersensitivity reactions is thought to be the HLA system. HLA class I alleles are expressed on all nucleated cells. HLA is the most polymorphic region of the human genome that has played a major evolutionary role in adaptive immune responses through presentation of foreign peptides to T cell receptors that in the case of an HLA-class I restricted response leads to activation of CD8^+^ T cells.^31^ Genetic variation in the HLA region alters the shape of the peptide-binding pocket in HLA molecules and enables their binding to a vast number of different peptides—a crucial step in the adaptive immune response.^32^ However, this ability of HLA molecules to bind a wide variety of peptides may also facilitate binding of exogenous molecules such as drugs, potentially leading to off-target drug effects and immune-mediated ADRs.^33^ The precise mechanism of most HLA-drug interactions remains unknown, but it seems that T cell activation is necessary for the majority of HLA-mediated ADRs.^33^, ^34^, ^35^ Despite the increasing evidence for a role of the HLA system in drug-induced hypersensitivity, much is still unclear mechanistically as to how genetic variation in the HLA region predisposes to specific drug reactions.

Penicillin is the most common cause of drug allergy, with clinical manifestations ranging from relatively benign cutaneous reactions to life-threatening systemic syndromes.^7^^,^^8^ There is a previous GWAS on the immediate type of penicillin allergy, where a borderline genome-wide significant protective association of an allele of the MHC class II gene *HLA-DRA* was detected and further replicated in a different cohort.^36^ Here we detect a robust association between self-reported penicillin allergy and an allele of the MHC class I gene *HLA-B*. The allele and its tag-SNP were also associated with lower lymphocyte counts and overlapped with T cell regulatory annotations. This raises the possibility that the variant may predispose to a T cell-mediated process that could lead to a delayed penicillin reaction through a heterologous response from an HLA-B^∗^55:01 restricted immune response that occurred earlier in life to a prevalent pathogen or an infection or disease interaction. MHC I molecules are expressed by almost all cells and present peptides to cytotoxic CD8^+^ T cells, whereas MHC II molecules are expressed by antigen-presenting cells to present peptides to CD4^+^ T helper lymphocyte.^32^^,^^35^ There are several examples of MHC I alleles associated with drug-induced hypersensitivity mediated by CD8^+^ T cells.^35^^,^^37^^,^^38^ The involvement of T cells in delayed hypersensitivity reactions has been shown by isolating drug-reactive T cell clones,^39^ and cytotoxic CD8^+^ T cells have been shown to be relevant especially in allergic skin reactions.^40^, ^41^, ^42^ More than 20 years ago, CD8^+^ T cells reactive to penicillin were isolated from patients with delayed type of hypersensitivity to penicillin.^43^ The association with the HLA-B^∗^55:01 allele detected in our study might be a relevant factor in this established connection with CD8^+^ T cells as HLA-B07-supertype alleles that share peptide binding specificities with HLA-B^∗^55:01 have previously been associated with nevirapine-induced rash.^44^ The underlying mechanism in penicillin allergy remains a question and various models have been proposed for T cell-mediated hypersensitivity.^37^^,^^42^ For example, the hapten model suggests that drugs may alter proteins and thereby induce an immune response^37^^,^^45^—penicillins have been shown to bind proteins^45^^,^^46^ to form hapten-carrier complexes, which may in turn elicit a T cell response.^47^ Drugs may also non-covalently interact with MHC molecules and alter the repertoire of bound peptides leading to presentation of antigens to which the host has not been previously tolerized. For example, abacavir has been shown to bind non-covalently within the F pocket of the antigen binding cleft of HLA-B^∗^57:01, altering its peptide specificity and leading to a CD8^+^ T cell-mediated hypersensitivity response.^48^, ^49^, ^50^

It is increasingly recognized that the involvement of HLA variation in hypersensitivity reactions goes beyond peptide specificity. Other factors, such as effects on HLA expression that influence the strength of the immune response, have also been described.^51^ The analysis of eQTLs based on the data of the eQTLGen Consortium^22^ revealed that the lead SNP rs114892859 identified in our GWAS of penicillin allergy appears to be associated with the expression of several nearby genes, including expression of both *HLA-B* and *HLA-C*, and an even stronger effect on RNA levels of *PSORS1C3* and *MICA* (Table S2). Variants in *PSORS1C3* have been associated with the risk of allopurinol-, carbamazepine-, and phenytoin-induced SJS/TEN hypersensitivity reactions^52^ and *MICA* encodes the protein MHC class I polypeptide-related sequence A^53^ which has been implicated in immune surveillance.^54^^,^^55^ Our findings therefore support the observation that variants associated with expression of HLA genes may contribute to the development of hypersensitivity reactions.

We also detected an association with variants in *PTPN22* on chromosome 1. *PTPN22* encodes a tyrosine phosphatase involved in the regulation of immune cell signaling.^56^ The lead missense variant rs2476601 has previously been associated with several autoimmune diseases^57^ and is a risk allele for rheumatoid arthritis.^58^ Interestingly, this variant was also recently shown to be associated with drug-induced liver injury (DILI).^59^ The association with the rs2476601 variant was strongest for cases of amoxicillin- and clavulanic acid-associated liver injury (OR 1.62, p value 4.0 × 10^−6^). Case subjects in this study were clinically sourced and comprehensively phenotyped, which suggests that our self-reported penicillin allergy phenotype might also capture signal related to more severe forms of beta-lactam hypersensitivity. However, the effect of this variant on penicillin allergy in the current study is relatively small (OR 1.09) and its role in the development of allergic reactions needs further studies.

A genetic correlation analysis of the penicillin allergy GWAS results in the current study revealed overlap with rheumatoid arthritis, even when excluding the *PTPN22* region from the analysis. Furthermore, we identified a genetic correlation with psoriasis, another autoimmune disease. Both psoriasis and psoriatic arthritis also have associations with *HLA-B* alleles.^60^^,^^61^ This indicates a possible underlying autoimmune factor in the development of the penicillin allergy phenotype investigated in our study.

Studies have suggested that penicillin allergy labels are acquired in childhood and of children labeled as penicillin allergic, 75% have acquired this label by age 3.^8^ In addition, approximately 10% of patients tested per year will lose their skin test reactivity, meaning that by adulthood >95% of allergy labels can be removed with formal testing. The main limitation of this study is the unverified nature of the phenotypes extracted from EHRs and self-reported data in the biobanks. Previous work has found that most (90%–95%) individuals labeled as having beta-lactam hypersensitivity may not actually have true hypersensitivity by adulthood when they more commonly undergo validated testing.^7^^,^^8^^,^^62^^,^^63^ However, we believe that the phenotype we have studied is valid for several reasons.^7^^,^^62^ The most commonly reported penicillin allergy is delayed-type allergy, which usually manifests as a transient benign rash that does not recur on rechallenge many years later.^7^^,^^62^^,^^64^ Furthermore, many individuals who were once labeled as having IgE-mediated penicillin allergy develop tolerance over time.^7^ In both of these cases, a true penicillin-induced reaction occurred initially, even if tolerance develops subsequently. Our phenotype therefore could represent individuals that experienced a reaction associated to penicillin when they were previously exposed, but who may, over time, tolerate penicillin administration. A delayed rash is the most common self-reported reaction seen in association with penicillin,^7^^,^^62^^,^^64^ which is frequently a T cell-mediated process. The results from our *in silico* analyses, which link HLA-B^∗^55:01 to T cell biology, would support this observation. Therefore, we posit that the association with the HLA-B^∗^55:01 allele may represent a predisposition to an immune response associated with penicillin which does not appear to be associated with a severe immediate or delayed reaction and which may wane with time.

Despite the possibility that some cases in our study may be misclassified, we detect a robust HLA association that was replicated in several independent cohorts against related phenotypes. The increased power arising from biobank-scale sample sizes therefore mitigates some of the challenges associated with EHR data. The robustness of the genetic signal across cohorts with orthogonal phenotyping methods, ranging from EHR-sourced in EstBB and BioVU to various forms of self-reported data in UKBB and 23andMe, also supports a true association. Finally, the modest effect size of the HLA-B^∗^55:01 allele (OR 1.33), particularly when compared to effect sizes of HLA alleles with established pharmacogenetic relevance,^65^, ^66^, ^67^ suggests that this variant has limited predictive value. However, further phenotypic refinement, including investigation of specific penicillin derivatives and specific types of drug reactions, may yield more clinically actionable insight.

In summary, we have leveraged data from four large-scale cohorts, including more than 100,000 case subjects, to provide insights into the genetic architecture of self-reported penicillin allergy and to provide robust evidence implicating the HLA-B^∗^55:01 allele in this condition. Further studies are necessary to determine the precise underlying immune processes and how these change over time.

## Consortia

The members of 23andMe research team: Michelle Agee, Stella Aslibekyan, Robert K. Bell, Katarzyna Bryc, Sarah K. Clark, Sarah L. Elson, Kipper Fletez-Brant, Pierre Fontanillas, Nicholas A. Furlotte, Pooja M. Gandhi, Karl Heilbron, Barry Hicks, David A. Hinds, Karen E. Huber, Ethan M. Jewett, Yunxuan Jiang, Aaron Kleinman, Keng-Han Lin, Nadia K. Litterman, Marie K. Luff, Jennifer C. McCreight, Matthew H. McIntyre, Kimberly F. McManus, Joanna L. Mountain, Sahar V. Mozaffari, Priyanka Nandakumar, Elizabeth S. Noblin, Carrie A.M. Northover, Jared O’Connell, Aaron A. Petrakovitz, Steven J. Pitts, G. David Poznik, J. Fah Sathirapongsasuti, Anjali J. Shastri, Janie F. Shelton, Suyash Shringarpure, Chao Tian, Joyce Y. Tung, Robert J. Tunney, Vladimir Vacic, Xin Wang, and Amir S. Zare.

## Author Contributions

K.K., L.M., and J.F. designed the study. R.M., M.L., Y.L., S.R., E.J.P., D.M.R., W.-Q.W., A.M., and T.E. supervised and generated genotype data or HLA typing data. D.S. and S.L. generated allergy data from free text. K.K., J.B., N.Z., M.L., T.J., J.C.C., T.L., J.F., W.W., and A.A. performed the data analysis. E.A. conducted amino acid sequence analysis. K.K., J.B., N.Z., M.V.H., C.M.L., R.M., L.M., J.C.C., E.J.P., W.-Q.W., and J.F. conducted data interpretation. K.K. prepared the figures and tables. K.K., J.B., L.M., and J.F. drafted the manuscript. K.K., J.B., N.Z., M.V.H., C.M.L., M.L., R.M., L.M., J.C.C., W.W., A.A., E.J.P., J.F., B.F., F.G., and L.S. reviewed and edited the manuscript. All authors contributed to critical revisions and approved the final manuscript.

The corresponding author attests that all listed authors meet authorship criteria and that no others meeting the criteria have been omitted.

## Declaration of Interests

C.M.L. has collaborated with Novo Nordisk and Bayer in research, and in accordance with a university agreement, did not accept any personal payment. W.W., A.A., and members of the 23andMe Research Team are employed by and hold stock or stock options in 23andMe, Inc. There were no other relationships or activities that could appear to have influenced the submitted work. The views expressed are those of the author(s) and not necessarily those of the NHS, the NIHR, the Department of Health, or the NIH.

## Web Resources

Pan-UKB, https://pan.ukbb.broadinstitute.org
R statistical software, https://www.r-project.org/
UK Biobank: Resource 182, https://biobank.ctsu.ox.ac.uk/crystal/refer.cgi?id=182

## Data and Code Availability

All code used for the analysis is described in the Subjects and Methods section of the manuscript. Genotype and phenotype data are available from the Estonian Biobank (https://genomics.ut.ee/en/biobank.ee/data-access) and UK Biobank (https://www.ukbiobank.ac.uk/using-the-resource/) upon request.

## Supplemental Data

Document S1. Figures S1–S4, Tables S3, S7, and S9, and Supplemental Subjects and Methods

Table S1 Genome-wide Significant Associations of Meta-analysis of Penicillin Allergy

Table S2 Associations of Expression Quantitative Trait Locus (eQTL) in Blood with the Results of Penicillin Allergy Meta-analysis Based on the eQTLGen Consortium Data eQTLGen is a meta-analysis of cis-/trans-eQTLs from 37 datasets with a total of 31,684 individuals. Signed stats column indicates the direction that is either “+” indicating that risk increasing allele increases the expression of the gene or “-” indicating the risk increasing allele decreases the expression of the gene.

Table S4 The Frequencies of HLA Four-Digit Alleles in Estonian and UK Biobank

Table S5 The Frequency Difference Test between European versus Asian and European versus African Populations using Wilcoxon Test for All HLA Alleles

Table S6 Summary Statistics of Associations between Penicillin Allergy and Four-Digit HLA Haplotypes in Estonian, UK, and BioVU Biobank

Table S8 Amino Acid Sequence Similarity of 48 HLA-B Allele Serotypes Present in Estonian and UK Biobanks Cells Green and yellow coloring indicates the similarity between values within columns (%), while blue and red coloring indicate a binary amino acid conservation within the specific column.

Document S2. Article plus Supplemental Information
