# Supplementary material for: Genome-wide Study Identifies Association between HLA-B∗55:01 and Self-Reported Penicillin Allergy
Source: Am J Hum Genet. 2020 Sep 3;107(4):612–21. doi: 10.1016/j.ajhg.2020.08.008 (PMC7536643; doi:10.1016/j.ajhg.2020.08.008)
Supplement: Document S1. Figures S1–S4, Tables S3, S7, and S9, and Supplemental Subjects and Methods [file mmc1.pdf]

The American Journal of Human Genetics, Volume *107*

Supplemental Data

Genome-wide Study Identifies Association between HLA-B*55:01 and Self-Reported Penicillin Allergy

Kristi Krebs, Jonas Bovijn, Neil Zheng, Maarja Lepamets, Jenny C. Censin, Tuuli Jürgenson, Dage Särg, Erik Abner, Triin Laisk, Yang Luo, Line Skotte, Frank Geller, Bjarke Feenstra, Wei Wang, Adam Auton, 23andMe Research Team, Soumya Raychaudhuri, Tõnu Esko, Andres Metspalu, Sven Laur, Dan M. Roden, Wei-Qi Wei, Michael V. Holmes, Cecilia M. Lindgren, Elizabeth J. Phillips, Reedik Mägi, Lili Milani, and João Fadista

Supplemental Data

Supplemental Figures *3*

Figure S1. The quantile-quantile (QQ) plot for the genome-wide meta-analysis of self-reported penicillin allergy. The observed lambda value is 1.03. 3

Figure S2. The distribution of allele frequencies of MHC class I genes HLA-A, HLA-B and HLA-C in European (darker grey), Asian (lighter grey) and African (green) populations. The frequency of allels in Estonian (blue) and UK (red) biobanks are shown in respectively colored dots. 4

Figure S3. The distribution of allele frequencies of MHC class II genes HLA-DRB1, HLA-DQB1 and HLA-DQA1 in European (darker grey), Asian (lighter grey) and African (green) populations. The frequency of allels in Estonian (blue) and UK (red) biobanks are shown in respectively colored dots 5

Figure S4. HLA-B*55:01 exhibits structural differences from another common HLA-B allele. Yellow residues highlight the two different amino acids in the antigen-binding cleft between 55:01 (left) and 56:01 (right). 6

Supplemental Tables *7*

Table S1. Genome-wide significant associations of meta-analysis of penicillin allergy 7

Table S2. Associations of expression quantitative trait locus (eQTL) in blood with the results of penicillin allergy meta-analysis based on the eQTLGen Consortium data. eQTLGen is a meta-analysis of cis-/trans-eQTLs from 37 datasets with a total of 31,684 individuals. Signed stats column indicates the direction that is either "+" indicating that risk increasing allele increases the expression of the gene or "-" indicating the risk increasing allele decreases the expression of the gene 7

Table S3. Summary statistics of association of the rs114892859 variant with penicillin allergy in non-European ancestries based on the Pan-UKB database 8

Table S4. The frequencies of HLA four-digit alleles in Estonian and UK biobank. 9

Table S5. The frequency difference test between European vs Asian and European vs African populations using Wilcoxon test for all HLA alleles. 9

Table S6. Summary statistics of associations between penicillin allergy and four-digit HLA haplotypes in Estonian, UK and BioVU biobank. 9

Table S7. The HLA-B*5501 allele correlation with the SNPs in the HLA region in Estonian and UK biobank 10

Table S8. Amino acid sequence similarity of 48 HLA-B allele serotypes present in Estonian and UK biobanks. Cells Green and yellow colouring indicates the similarity between values within columns (%), while blue and red colouring indicate a binary amino acid conservation within the specific column. 11

Table S9. Genetic correlation of self-reported penicillin allergy with published autoimmune and hematological traits using LDHub. PMID – PubMed ID (reference study); rg- genetic correlation; se- standard error of rg; z- z-score of rg; p- p-value of rg. 12

Supplemental Methods *13*

Phenotype definitions 13

Genotype information 14

Genome-wide study of penicillin allergy 16

Post-GWAS annotation 16

HLA typing 17

Comparison of HLA allele frequencies 18

Replication in 23andMe 19

HLA-B*55:01 allele association with lymphocyte levels in EstBB 19

Comparison of the amino acid sequences of HLA-B alleles 20

Supplemental Acknowledgements 20

Supplemental References 21

Supplemental Figures

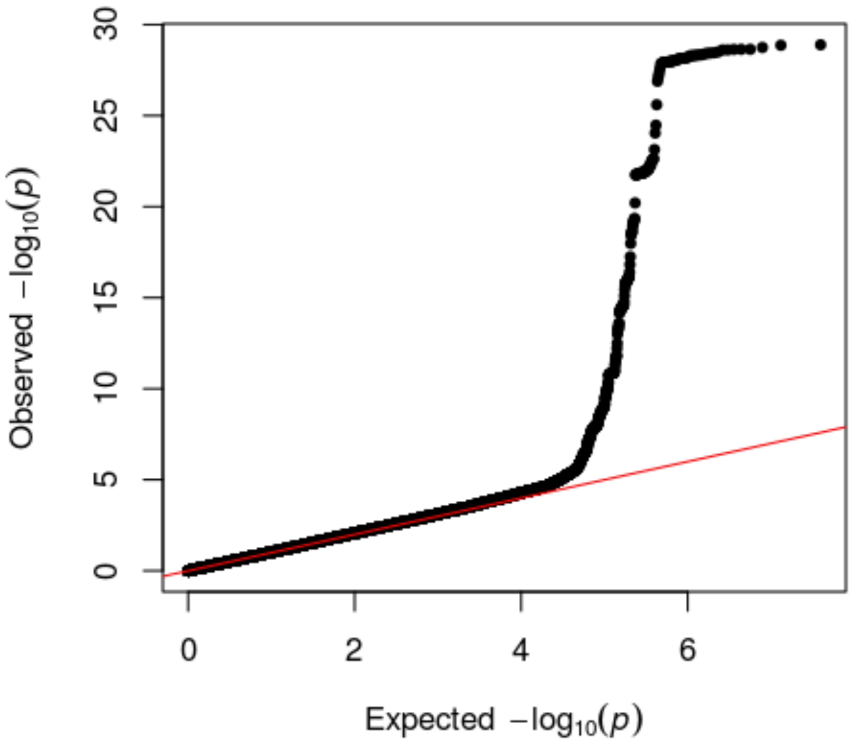

Figure S1. The quantile-quantile (QQ) plot for the genome-wide meta-analysis of self-reported penicillin allergy. The observed lambda value is 1.03.

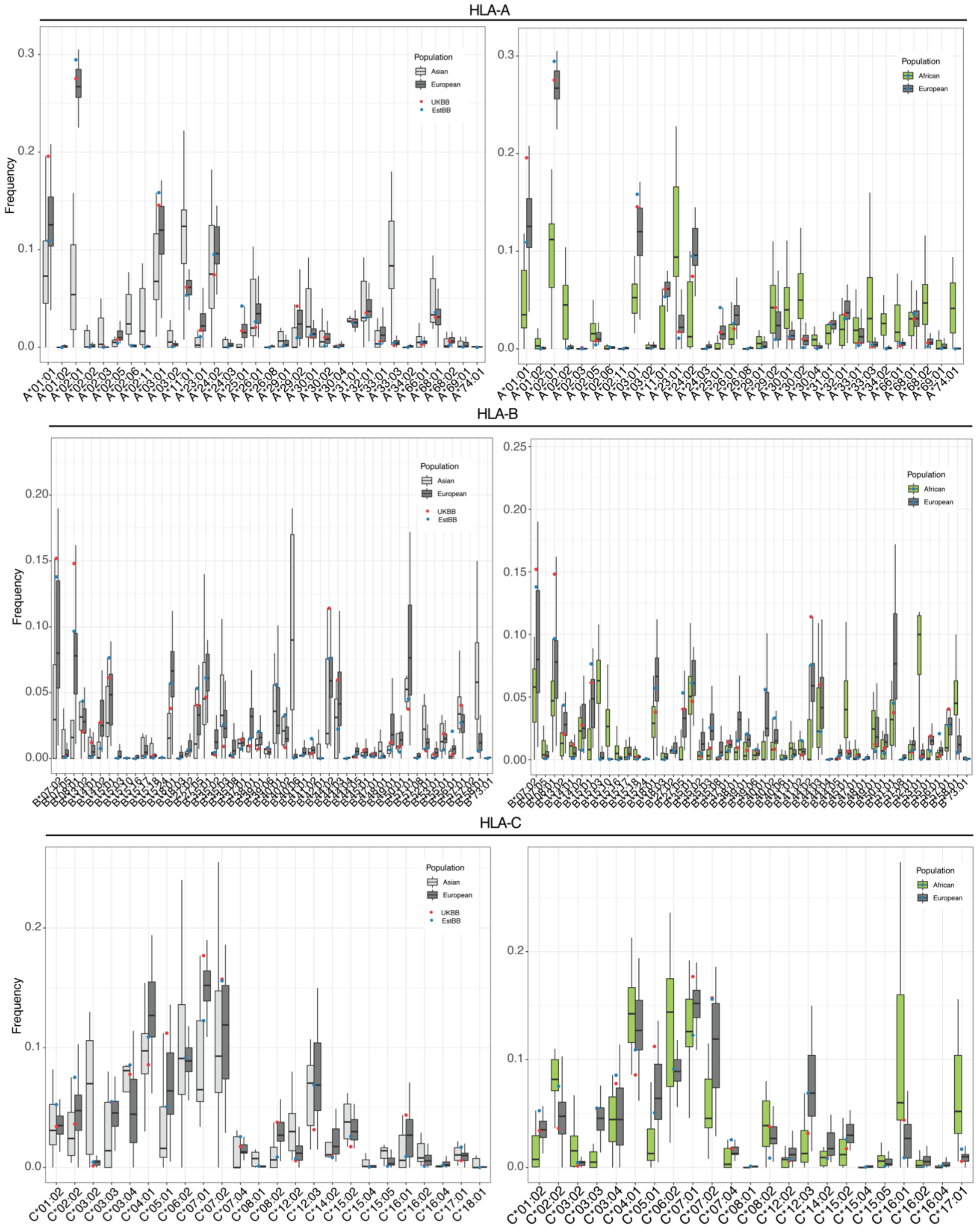

Figure S2. The distribution of allele frequencies of MHC class I genes HLA-A, HLA-B and HLA-C in European (darker grey), Asian (lighter grey) and African (green) populations**.** The frequency of allels in Estonian (blue) and UK (red) biobanks are shown in respectively colored dots.

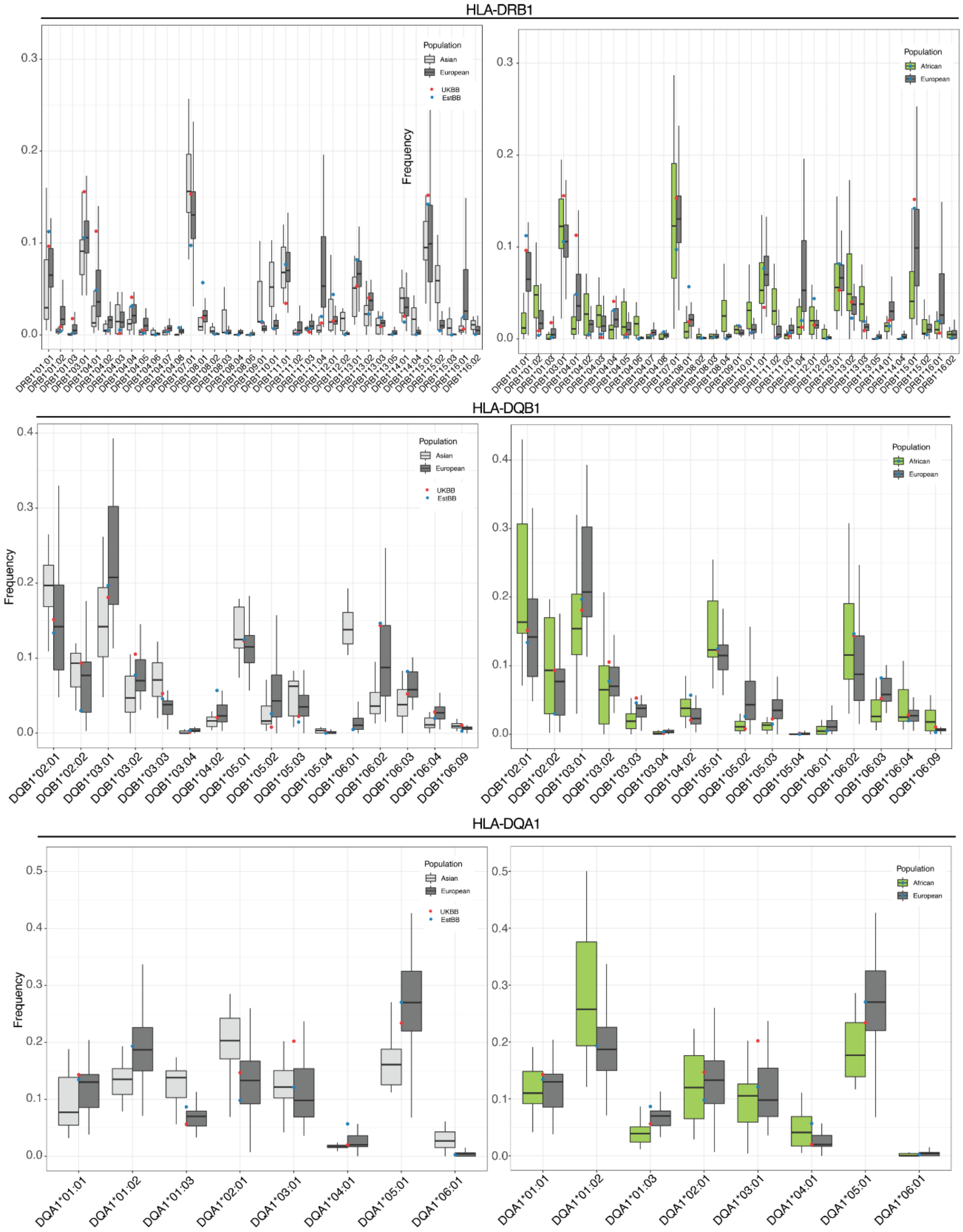

Figure S3. The distribution of allele frequencies of MHC class II genes HLA-DRB1, HLA-DQB1 and HLA-DQA1 in European (darker grey), Asian (lighter grey) and African (green) populations**.** The frequency of allels in Estonian (blue) and UK (red) biobanks are shown in respectively colored dots.

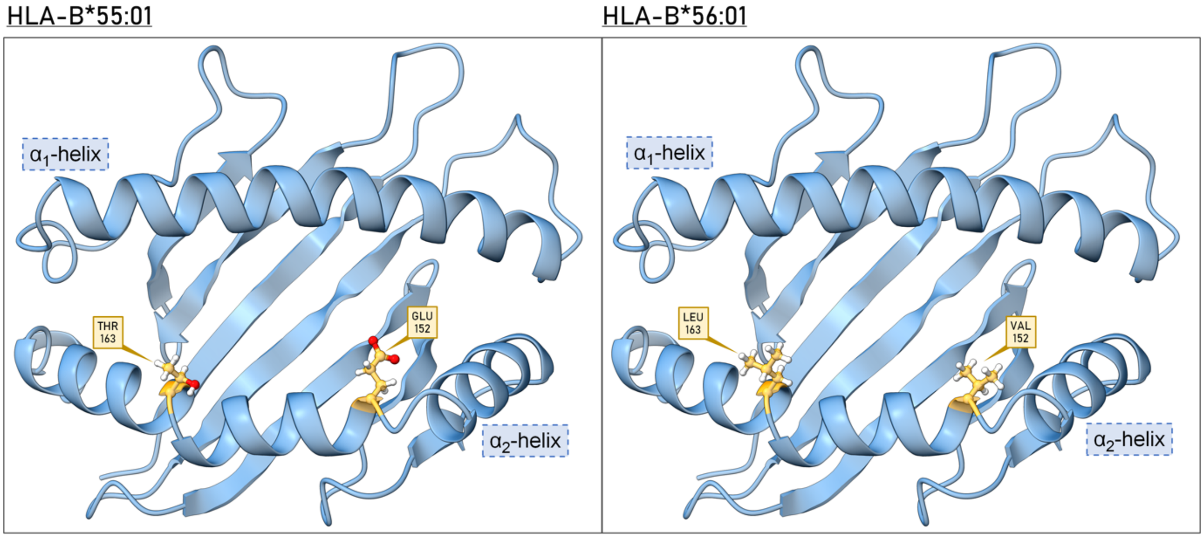

Figure S4. HLA-B*55:01 exhibits structural differences from another common HLA-B allele. Yellow residues highlight the two different amino acids in the antigen-binding cleft between 55:01 (left) and 56:01 (right).

Supplemental Tables

Table S1. Genome-wide significant associations of meta-analysis of penicillin allergy

Table S2. Associations of expression quantitative trait locus (eQTL) in blood with the results of penicillin allergy meta-analysis based on the eQTLGen Consortium data. eQTLGen is a meta-analysis of cis-/trans-eQTLs from 37 datasets with a total of 31,684 individuals. Signed stats column indicates the direction that is either "+" indicating that risk increasing allele increases the expression of the gene or "-" indicating the risk increasing allele decreases the expression of the gene.

Allergy/adverse effect of penicillin in PanUKB database	
**Chromosome**	6
**Position**	31369278
**rsID**	rs114892859
**Ref allele**	G
**Alt allele**	T
**European ancestry**	
**N cases**	20,021
**N Controls**	383,239
**Allele frequency of cases**	0.027
**Allele frequency of controls**	0.018
**Beta**	0.486
**Standard error**	0.041
**P-value**	$6,16 \times 10^{-33}$
**African ancestry**	
**N cases**	298
**N Controls**	6,089
**Allele frequency of cases**	0.000
**Allele frequency of controls**	0.002
**Beta**	-1.081
**Standard error**	1.041
**P-value**	0.299
**Central/South Asian ancestry**	
**N cases**	434
**N Controls**	8,150
**Allele frequency of cases**	0.020
**Allele frequency of controls**	0.022
**Beta**	-0.114
**Standard error**	0.239
**P-value**	0.633
**East Asian ancestry**	
**N cases**	93
**N Controls**	2,535
**Allele frequency of cases**	NA
**Allele frequency of controls**	NA
**Beta**	NA
**Standard error**	NA
**P-value**	NA
**Middle Eastern ancestry**	
**N cases**	72
**N Controls**	1,478
**Allele frequency of cases**	0.035
**Allele frequency of controls**	0.021
**Beta**	0.311
**Standard error**	0.549
**P-value**	0.571

Table S3. Summary statistics of association of the rs114892859 variant with penicillin allergy in non-European ancestries based on the Pan-UKB database

Table S4. The frequencies of HLA four-digit alleles in Estonian and UK biobank.

Table S5. The frequency difference test between European vs Asian and European vs African populations using Wilcoxon test for all HLA alleles.

Table S6. Summary statistics of associations between penicillin allergy and four-digit HLA haplotypes in Estonian, UK and BioVU biobank.

Chromosome	Location	SNP	r_EstBB	r_UKBB
6	31369278	rs114892859	0.98	0.96
6	31374671	rs144626001	0.98	0.97
6	31352678	rs183120114	0.84	0.75
6	31371342	rs2301750	0.58	0.78
6	31326140	rs114492969	0.49	0.78
6	31325201	rs74194187	0.49	0.78
6	31326099	rs114734598	0.49	0.78
6	31326157	6_31326157	0.49	0.78
6	31326312	rs145887584	0.49	0.78
6	31326959	rs114166883	0.49	0.78
6	31327114	rs72502572	0.49	0.78
6	31327265	rs114783056	0.49	0.79
6	31327446	rs72502573	0.49	0.78
6	31327622	rs115200108	0.49	0.80
6	31336558	rs60177449	0.49	0.80
6	31342532	rs147336204	0.49	0.80
6	31344484	rs114654060	0.49	0.80
6	31344485	rs116355076	0.49	0.80
6	31348200	rs75931194	0.49	0.80
6	31334799	rs7766461	0.49	0.79
6	31321657	rs3177747	0.49	0.77
6	31321845	rs361531	0.49	0.77
6	31322767	rs3819284	0.49	0.77
6	31323414	rs41563818	0.49	0.70
6	31323707	rs41545339	0.49	0.71
6	31351321	rs72865324	0.49	0.80
6	31355813	rs2428484	0.49	0.80
6	31353285	rs72882965	0.49	0.80
6	31326178	rs184227609	0.48	0.70
6	31340628	rs72878037	0.48	0.80
6	31327326	rs68085422	0.48	0.79
6	31340795	rs72878039	0.48	0.79
6	31274734	rs114411923	0.34	0.28
6	31324764	rs41560522	0.30	0.41
6	31271783	rs145970926	0.29	0.27
6	31360838	rs142740116	0.29	0.27
6	31360289	rs72867732	0.29	0.27
6	31366969	rs72883110	0.28	0.27
6	31367052	rs146482045	0.28	0.27
6	31364848	rs72502580	0.28	0.29
6	31363312	rs140766555	0.28	0.22
6	31352694	rs56671953	0.27	0.26
6	31354939	rs72882972	0.27	0.26
6	31321184	rs72866766	0.22	0.25

Table S7. The HLA-B*5501 allele correlation with the SNPs in the HLA region in Estonian and UK biobank.

Table S8. Amino acid sequence similarity of 48 HLA-B allele serotypes present in Estonian and UK biobanks. Cells Green and yellow colouring indicates the similarity between values within columns (%), while blue and red colouring indicate a binary amino acid conservation within the specific column.

Trait	PMID	Category	Ethnicity	rg	se	z	p
Rheumatoid Arthritis	24390342	autoimmune	European	0.3531	0.0694	5.0866	3.65E-07
Asthma	17611496	autoimmune	European	0.3745	0.1235	3.0313	0.0024
Systemic lupus erythematosus	26502338	autoimmune	European	0.2182	0.1024	2.1321	0.0330
Multiple sclerosis	21833088	autoimmune	European	0.3558	0.1805	1.9713	0.0487
Primary biliary cirrhosis	26394269	autoimmune	European	0.1905	0.0971	1.9623	0.0497
Eczema	26482879	autoimmune	Mixed	0.2234	0.1219	1.8323	0.0669
Mean platelet volume	22139419	haemotological	European	0.1501	0.092	1.6311	0.1029
Heart rate	23583979	haemotological	Mixed	0.1117	0.0707	1.5804	0.1140
Inflammatory Bowel Disease (Euro)	26192919	autoimmune	European	0.079	0.0684	1.1552	0.2480
Crohns disease	26192919	autoimmune	European	0.0698	0.0712	0.9795	0.3273
Celiac disease	20190752	autoimmune	European	0.1049	0.1109	0.946	0.3441
Ulcerative colitis	26192919	autoimmune	European	0.0526	0.0777	0.6765	0.4987
Primary sclerosing cholangitis	27992413	autoimmune	Mixed	0.0104	0.0899	0.1157	0.9079
Platelet count	22139419	haemotological	European	-0.0084	0.0797	-0.1051	0,9163

Table S9. Genetic correlation of self-reported penicillin allergy with published autoimmune and hematological traits using LDHub. PMID – PubMed ID (reference study); rg- genetic correlation; se- standard error of rg; z- z-score of rg; p- p-value of rg.

Supplemental Methods

Phenotype definitions

All participants in both UK and Estonian Biobanks signed a consent form to allow follow-up linkage of their electronic health records (EHR), thereby enabling longitudinal collection of phenotypic information. EstBB allows access to the records of the national Health Insurance Fund Treatment Bills (since 2004), Tartu University Hospital (since 2008), and North Estonia Medical Center (since 2005). For every participant there is information on diagnoses in ICD-10 coding and drug dispensing data, including drug ATC codes, prescription status and purchase date (if available).

We extracted information on penicillin allergy by searching the records of the participants for Z88.0 ICD10 code. However, since the Z88.0 code seemed underreported in Estonia, we also used self-reported data on side-effects from penicillin for participants who reported hypersensitivity due to J01C* ATC drug group (Beta-Lactam Antibacterials, Penicillins) in their EstBB enrolment questionnaire. To validate this approach in EstBB we analyzed the effect of self-reported allergy status on the number on penicillin prescriptions in EstBB. We performed a Poisson regression among 37,825 unrelated individuals with J01C* prescriptions considering age, gender and 10 principal components (PC) as covariates. Units were interpreted as follows: 1-exp(beta)*100%=1-exp(-0.18)*100%= 16%. The Poisson model was considered appropriate as there was no large overdispersion.

To extract penicillin allergy from free-text we used a rule-based approach; the text had to contain any of the possible forms of the words ‘allergy’ or ‘allergic’ in Estonian as well as a potential variation of a penicillin name. As drug names are often misspelled, abbreviated or written using the English or Latin spelling instead of the standard

Estonian one, we used a regular expression to capture as many variations of each penicillin name as possible. In addition, we applied rules regarding the distance between the words ‘allergy’ and the drug name as well as other words nearby to exclude negations of penicillin allergies in the definition. This together with questionnaire data resulted in 1,320 cases with penicillin allergy.

For BioVU, penicillin allergies were extracted from the allergy sections of the clinical notes, which are often used to document a patient’s intolerance or allergy to a drug as reported by the patient or observed by a healthcare provider.[1] The data in an allergy section in the clinical notes are semi-structured (e.g. penicillin [rash]). We defined penicillin allergy cases as individuals with any mention of the penicillin in the allergy section. Mentions of penicillin in the allergy section are identified using case-insensitive regular expressions that matched keywords for generic names, brand names, abbreviations (e.g., pcn), and common misspellings.

Genotype information

In brief, the 51,936 EstBB participants have been genotyped using the Global Screening Array v1 (GSA). Individuals were excluded from the analysis if their call-rate was < 95% or sex defined based on heterozygosity of X chromosome did not match sex in phenotype data. Variants were filtered by call-rate < 95% and HWE p-value < 1e-4 (autosomal variants only). Variant positions were updated to b37 and all variants were changed to be from TOP strand using tools and reference files provided in https://www.well.ox.ac.uk/~wrayner/strand/ webpage. Before imputation variants with MAF<1% and indels were removed. Phasing was done using Eagle v2.3

software[2] (number of conditioning haplotypes Eagle2 uses when phasing each sample was set to: --Kpbwt=20000) and imputation was done using Beagle v.28Sep18.793[3] using the Estonian population specific imputation reference panel constructed of 2,297 whole genome sequenced samples.

In UKBB genotype data are available for 488,377 participants of which 49,950 are genotyped using the Applied Biosystems™ UK BiLEVE Axiom™ and the remaining 438,427 individuals were genotyped using the Applied Biosystems™ UK Biobank Axiom™ Array by Affymetrix. The genotype data was phased using SHAPEIT3[4], and imputation was conducted using IMPUTE4[5] using a combined version of the Haplotype Reference Consortium (HRC) panel[6] and the UK10K panel.[7] We excluded individuals who have withdrawn their consent, have been labelled by UKBB to have poor heterozygosity or missingness, who have putative sex chromosome aneuploidy and who have >10 relatives in the dataset. We further removed all individuals with mismatching genetic and self-reported sex and ethnicity. GWAS was executed on individuals with confirmed white British ancestry.

Genotyping in Vanderbilt University Medical Center BioVU DNA Biobank was performed on the Infinium Multi-Ethnic Genotyping Array (MEGAchip). We excluded DNA samples: (1) with per-individual call rate < 95%; (2) with wrongly assigned sex; or (3) unexpected duplication. We performed whole genome imputation using the Michigan Imputation Server (https://imputationserver.sph.umich.edu)[8] with the Haplotype Reference Consortium, version r1.1[6], as reference. Principle components for ancestry (PCs) were calculated using common variants (MAF > 0.01) with high

variant call rate (> 98%), excluding variants in linkage and regions known to affect PCs (HLA region on chromosome 6, inversion on chromosome 8 (8135000-12000000) and inversion on chr 17 (40900000-45000000), GRCh37 build). For association analyses, we used EasyQC (www.genepi-regensburg.de/easyqc)[9] to filter (1) poorly imputed variants with imputation info r^2 value of < 0.5, (2) MAF < 0.005, (3) deviation from Hardy-Weinberg equilibrium with a P-value ≤ 1×10^{-6} and (4) variants with MAF that deviated from the HRC reference panel by > 0.3.

Genome-wide study of penicillin allergy

Using the SAIGE software[10], we applied generalized mixed models with saddlepoint approximation to account for case-control imbalance and relatedness in all three cohorts. In EstBB the controls were selected from a set of individuals with no self-reported ADRs or with ICD10 diagnoses covered in a list of 79 ICD10 codes (described in [11]) with a possible drug-induced nature or diagnoses described as “due to drugs”. To minimize the effects of population admixture and stratification, the analyses only included samples with European ancestry based on PC analysis (PCA) and were adjusted for the first 10 PCs of the genotype matrix, as well as for birthyear and sex. In UKBB similarly as for EstBB, the GWAS was adjusted for the first 10 PCs of the genotype matrix, as well as for age and sex. In BioVU regression models were adjusted for sex, age, EHR length (years), and the first 10 principle components of the genotyping array for ancestry.

Post-GWAS annotation

FUMA (Functional mapping and annotation of genetic associations)[12] is an integrative web-based platform using information from multiple biological resources, including e.g. information on eQTLs, chromatin interaction mappings, and LD structure to annotate GWAS data. We applied FUMA to identify lead SNPs and genomic risk loci for results of the meta-analysis, using the European LD reference panel from 1000G.[13] Further eQTL associations were identified based on data from the eQTLGen consortium, which is a meta-analysis of 37 datasets with blood gene expression data pertaining to 31,684 individuals.[14]

HaploReg[15] was used for exploring annotations, chromatin states, conservation, and regulatory motif alterations. To estimate the relative deleteriousness of the identified SNPs, we used the Combined Annotation Dependent Depletion (CADD) framework.[16]

HLA typing

The SNP2HLA tool imputes HLA alleles from SNP genotype data and single Nucleotide Variants (SNVs), small INsertions and DELetions (INDELs) and classical HLA variants were called using whole genome sequences of 2,244 study participants from the Estonian Biobank sequenced at 26.1x. We performed high-resolution (G-group) HLA calling of three class-I HLA genes (HLA-A, -B and -C) and three class-II HLA genes (HLA-DRB1, -DQA1 and -DQB1) using the HLA*PRG algorithm.[17] SNVs and INDELs were called using GATK version 3.6 according to the best practices for variant discovery.[18] Classical HLA alleles, HLA amino acid residues and untyped SNPs were then imputed using SNP2HLA and the reference panel constructed using the 2,244 whole-genome sequenced Estonian samples. We performed an additive

logistic regression analysis with the called HLA alleles using R *glm* function in EstBB including age, sex and 10 PCs as covariates.

In UKBB, for each genotype call one metric is reported, the absolute posterior probability of the allele inference. We applied thresholding to the maximum posterior probability (at a threshold of 0.8) to create a marker representing the presence/absence of each HLA allele for each individual participant. Only alleles with a minor allele frequency of > 0.01% were included in the analysis, amounting to 202 alleles taken forward for association testing with penicillin allergy. In UKBB we performed association analysis of each four-digit allele with the Z88.0 subcode using logistic regression function *glm* in R, adjusting for sex, age, age^2, recruitment center, genotyping array, and the first 15 principal components (and excluding one individual of each pair of related [up to 2nd degree or closer, using KING's kinship coefficient > 0.0884] individuals and those of reported non-white ancestry).

For BioVU, SNP2HLA was used to impute four-digit HLA A B C DP DR DQ typing from SNP data from the MEGAchip. We performed an additive logistic regression analysis with the called HLA alleles using R *glm* function in BioVU including age, sex, EHR length (years), and 10 PCs as covariates.

Comparison of HLA allele frequencies

To compare obtained frequencies of HLA alleles with reported frequencies in European, Asian and African populations we used the database of Allele Frequencies of worldwide populations (http://www.allelefrequencies.net/default.asp). We queried

the frequencies of four-digit alleles choosing the following regions: Europe, North-East Asia, South-Asia, South-East Asia, Western Asia, North Africa and Sub-Saharan Africa. Frequency comparisons were visualized with R software (3.3.2)[19] using ggplot2 package and frequency difference was calculated with two-samples Wilcoxon test.

Replication in 23andMe

All individuals included in the analyses provided informed consent and participated in the research online, under a protocol approved by the external AAHRPP-accredited IRB, Ethical & Independent Review Services (E&I Review). Penicillin allergy was determined based on the survey questions for allergic symptoms or for questions related to allergy test. Survey questions for positive allergy test were "Have you ever had a positive allergy test to any of these medications? [CONCEPT: penicillin]"; or "Has a doctor confirmed that you had an allergic reaction to penicillin, amoxicillin, or ampicillin?". A logistic regression assuming an additive model for allelic effects was used with adjusting for age, sex, indicator variables to represent the genotyping platforms and the first five genotype principal components. In the 23andMe replication study, the HLA imputation was performed by using HIBAG [20] with the default settings. We imputed allelic dosage for HLA-A, B, C, DPB1, DQA1, DQB1 and DRB1 loci at four-digit resolution[21].

HLA-B*55:01 allele association with lymphocyte levels in EstBB

To study the association between the HLA-B*55:01 allele and lymphocyte levels in EstBB, we extracted the information on measured lymphocyte levels (number of cells per nanoliter) from the free text fields of the medical history of 4,567 unrelated

individuals with genotype data. After removing outliers based on the values of any data points which lie beyond the extremes of the whiskers (values > 3.58 and < 0.26), a linear regression was performed using R software and with age and sex as covariates.

Comparison of the amino acid sequences of HLA-B alleles

The sequences for the common 48 HLA-B variants within EstBB and UKBB were acquired from the IPD-IMGT/HLA database,[22] which subsequently were aligned with NCBI Protein BLAST.[23] The molecular structures for HLA-B*55:01 and HLA-B*56:01 were created via SWISS-MODEL[24] and visualized with UCSF ChimeraX[25], developed by the Resource for Biocomputing, Visualization, and Informatics at the University of California, San Francisco, with support from National Institutes of Health R01-GM129325 and the Office of Cyber Infrastructure and Computational Biology, National Institute of Allergy and Infectious Diseases.

Supplemental Acknowledgements

Computation used the Oxford Biomedical Research Computing (BMRC) facility, a joint development between the Wellcome Centre for Human Genetics and the Big Data Institute supported by Health Data Research UK and the NIHR Oxford Biomedical Research Centre. Financial support was provided by the Wellcome Trust Core Award Grant Number 203141/Z/16/Z. The views expressed are those of the authors and not necessarily those of the NHS, the NIHR or the Department of Health.

Supplemental References

1. Zhou, L., Dhopeshwarkar, N., Blumenthal, K.G., Goss, F., Topaz, M., Slight, S.P., and Bates, D.W. (2016). Drug allergies documented in electronic health records of a large healthcare system. Allergy Eur. J. Allergy Clin. Immunol. *71*, 1305–1313.

2. Loh, P.R., Danecek, P., Palamara, P.F., Fuchsberger, C., Reshef, Y.A., Finucane, H.K., Schoenherr, S., Forer, L., McCarthy, S., Abecasis, G.R., et al. (2016). Reference-based phasing using the Haplotype Reference Consortium panel. Nat. Genet. *48*, 1443–1448.

3. Browning, S.R., and Browning, B.L. (2007). Rapid and accurate haplotype phasing and missing-data inference for whole-genome association studies by use of localized haplotype clustering. Am. J. Hum. Genet. *81*, 1084–1097.

4. O'Connell, J., Sharp, K., Shrine, N., Wain, L., Hall, I., Tobin, M., Zagury, J.F., Delaneau, O., and Marchini, J. (2016). Haplotype estimation for biobank-scale data sets. Nat. Genet. *48*, 817–820.

5. Bycroft, C., Freeman, C., Petkova, D., Band, G., Elliott, L.T., Sharp, K., Motyer, A., Vukcevic, D., Delaneau, O., O'Connell, J., et al. (2018). The UK Biobank resource with deep phenotyping and genomic data. Nature *562*, 203–209.

6. McCarthy, S., Das, S., Kretzschmar, W., Delaneau, O., Wood, A.R., Teumer, A., Kang, H.M., Fuchsberger, C., Danecek, P., Sharp, K., et al. (2016). A reference panel of 64,976 haplotypes for genotype imputation. Nat. Genet. *48*, 1279–1283.

7. Walter, K., Min, J.L., Huang, J., Crooks, L., Memari, Y., McCarthy, S., Perry, J.R.B., Xu, C., Futema, M., Lawson, D., et al. (2015). The UK10K project identifies rare variants in health and disease. Nature *526*, 82–89.

8. Das, S., Forer, L., Schönherr, S., Sidore, C., Locke, A.E., Kwong, A., Vrieze, S.I.,

Chew, E.Y., Levy, S., McGue, M., et al. (2016). Next-generation genotype imputation service and methods. Nat. Genet. *48*, 1284–1287.

9. Winkler, T.W., Day, F.R., Croteau-Chonka, D.C., Wood, A.R., Locke, A.E., Mägi, R., Ferreira, T., Fall, T., Graff, M., Justice, A.E., et al. (2014). Quality control and conduct of genome-wide association meta-analyses. Nat. Protoc. *9*, 1192–1212.

10. Zhou, W., Nielsen, J.B., Fritsche, L.G., Dey, R., Gabrielsen, M.E., Wolford, B.N., LeFaive, J., VandeHaar, P., Gagliano, S.A., Gifford, A., et al. (2018). Efficiently controlling for case-control imbalance and sample relatedness in large-scale genetic association studies. Nat. Genet. *50*, 1335–1341.

11. Tasa, T., Krebs, K., Kals, M., Mägi, R., Lauschke, V.M., Haller, T., Puurand, T., Remm, M., Esko, T., Metspalu, A., et al. (2019). Genetic variation in the Estonian population: pharmacogenomics study of adverse drug effects using electronic health records. Eur. J. Hum. Genet. *27*, 442–454.

12. Watanabe, K., Taskesen, E., Van Bochoven, A., and Posthuma, D. (2017). Functional mapping and annotation of genetic associations with FUMA. Nat. Commun. *8*, 1–11.

13. Auton, A., Abecasis, G.R., Altshuler, D.M., Durbin, R.M., Bentley, D.R., Chakravarti, A., Clark, A.G., Donnelly, P., Eichler, E.E., Flicek, P., et al. (2015). A global reference for human genetic variation. Nature *526*, 68–74.

14. Võsa, U., Claringbould, A., Westra, H.-J., Bonder, M.J., Deelen, P., Zeng, B., Kirsten, H., Saha, A., Kreuzhuber, R., Kasela, S., et al. (2018). Unraveling the polygenic architecture of complex traits using blood eQTL metaanalysis. BioRxiv 447367.

15. Ward, L.D., and Kellis, M. (2012). HaploReg: a resource for exploring chromatin states, conservation, and regulatory motif alterations within sets of genetically linked

variants. Nucleic Acids Res. *40*, D930–D934.

16. Rentzsch, P., Witten, D., Cooper, G.M., Shendure, J., and Kircher, M. (2019). CADD: predicting the deleteriousness of variants throughout the human genome. Nucleic Acids Res. *47*, D886–D894.

17. Dilthey, A.T., Gourraud, P.-A., Mentzer, A.J., Cereb, N., Iqbal, Z., and McVean, G. (2016). High-Accuracy HLA Type Inference from Whole-Genome Sequencing Data Using Population Reference Graphs. PLOS Comput. Biol. *12*, e1005151.

18. Broad Institute GATK | Germline short variant discovery (SNPs + Indels).

19. R Core Team (2018). R: a language and environment for statistical computing.

20. Zheng, X., Shen, J., Cox, C., Wakefield, J.C., Ehm, M.G., Nelson, M.R., and Weir, B.S. (2014). HIBAG - HLA genotype imputation with attribute bagging. Pharmacogenomics J. *14*, 192–200.

21. Tian, C., Hromatka, B.S., Kiefer, A.K., Eriksson, N., Noble, S.M., Tung, J.Y., and Hinds, D.A. (2017). Genome-wide association and HLA region fine-mapping studies identify susceptibility loci for multiple common infections. Nat. Commun. *8*, 1–13.

22. Robinson, J., Barker, D.J., Georgiou, X., Cooper, M.A., Flicek, P., and Marsh, S.G.E. (2020). IPD-IMGT/HLA Database. Nucleic Acids Res. *48*, D948–D955.

23. Protein BLAST: search protein databases using a protein query.

24. Grosdidier, A., Zoete, V., and Michielin, O. (2011). SwissDock, a protein-small molecule docking web service based on EADock DSS. Nucleic Acids Res. *39*, W270-7.

25. Goddard, T.D., Huang, C.C., Meng, E.C., Pettersen, E.F., Couch, G.S., Morris, J.H., and Ferrin, T.E. (2018). UCSF ChimeraX: Meeting modern challenges in visualization and analysis. Protein Sci. *27*, 14–25.
