# Supplementary material for: Genome-wide Study Identifies Association between HLA-B∗55:01 and Self-Reported Penicillin Allergy
Source: Am J Hum Genet. 2020 Sep 3;107(4):612–21. doi: 10.1016/j.ajhg.2020.08.008 (PMC7536643; doi:10.1016/j.ajhg.2020.08.008)
Supplement: Document S2. Article plus Supplemental Information [file mmc8.pdf]

ARTICLE

Genome-wide Study Identifies Association between HLA-B*55:01 and Self-Reported Penicillin Allergy

Kristi Krebs,[1,2,25] Jonas Bovijn,[3,4,25] Neil Zheng,[5,25] Maarja Lepamets,[1,2] Jenny C. Censin,[3,4] Tuuli Jürgenson,[1] Dage Särg,[6] Erik Abner,[1] Triin Laisk,[1] Yang Luo,[7,8,9,10,11] Line Skotte,[12] Frank Geller,[12] Bjarke Feenstra,[12] Wei Wang,[13] Adam Auton,[13] 23andMe Research Team,[13] Soumya Raychaudhuri,[7,8,9,10,11,14] Tõnu Esko,[1] Andres Metspalu,[1] Sven Laur,[6,15] Dan M. Roden,[5,16,21] Wei-Qi Wei,[5] Michael V. Holmes,[4,17,18,19,25] Cecilia M. Lindgren,[3,4,17,20,25] Elizabeth J. Phillips,[16,21,22,25] Reedik Mägi,[1,25] Lili Milani,[1,25,*] and João Fadista[12,23,24,25]

Summary

Hypersensitivity reactions to drugs are often unpredictable and can be life threatening, underscoring a need for understanding their underlying mechanisms and risk factors. The extent to which germline genetic variation influences the risk of commonly reported drug allergies such as penicillin allergy remains largely unknown. We extracted data from the electronic health records of more than 600,000 participants from the UK, Estonian, and Vanderbilt University Medical Center's BioVU biobanks to study the role of genetic variation in the occurrence of self-reported penicillin hypersensitivity reactions. We used imputed SNP to HLA typing data from these cohorts to further fine map the human leukocyte antigen (HLA) association and replicated our results in 23andMe's research cohort involving a total of 1.12 million individuals. Genome-wide meta-analysis of penicillin allergy revealed two loci, including one located in the HLA region on chromosome 6. This signal was further fine-mapped to the HLA-B*55:01 allele (OR 1.41 95% CI 1.33–1.49, p value 2.04×10^{-31}) and confirmed by independent replication in 23andMe's research cohort (OR 1.30 95% CI 1.25–1.34, p value 1.00×10^{-47}). The lead SNP was also associated with lower lymphocyte counts and *in silico* follow-up suggests a potential effect on T-lymphocytes at HLA-B*55:01. We also observed a significant hit in *PTPN22* and the GWAS results correlated with the genetics of rheumatoid arthritis and psoriasis. We present robust evidence for the role of an allele of the major histocompatibility complex (MHC) I gene *HLA-B* in the occurrence of penicillin allergy.

Introduction

Adverse drug reactions (ADRs) are common in clinical practice and are associated with high morbidity and mortality. A meta-analysis of prospective studies in the US revealed the incidence of serious ADRs to be 6.7% among hospitalized patients and the cause of more than 100,000 deaths annually.[1] In Europe, ADRs are responsible for 3.5% of all hospital admissions, with 10.1% of patients experiencing ADRs during hospitalization and 197,000 fatal cases per year.[2,3] In the US, the cost of a single ADR event falls between 1,439 to 13,462 USD.[4]

ADRs are typically divided into two types of reactions. Type A reactions are more predictable and related to the pharmacological action of a drug, whereas type B reactions are idiosyncratic, less predictable, largely dose independent, and typically driven by hypersensitivity reactions involving the immune system.[5] Although type B reactions are less frequent (<20%) than type A reactions, they tend to be more severe and more often lead to the withdrawal of a drug from the market.[6] One of the most common

[1]Estonian Genome Center, Institute of Genomics, University of Tartu, Tartu 51010, Estonia; [2]Institute of Molecular and Cell Biology, University of Tartu, Tartu 51010, Estonia; [3]Wellcome Centre for Human Genetics, Nuffield Department of Medicine, University of Oxford, Oxford OX3 7BN, UK; [4]Big Data Institute at the Li Ka Shing Centre for Health Information and Discovery, University of Oxford, Oxford OX3 7FZ, UK; [5]Department of Biomedical Informatics, Vanderbilt University Medical Center, Nashville, TN 37232, USA; [6]Institute of Computer Science, University of Tartu, Tartu 51009, Estonia; [7]Division of Rheumatology, Inflammation and Immunity, Brigham and Women's Hospital, Harvard Medical School, Boston, MA 02115, USA; [8]Division of Genetics, Brigham and Women's Hospital, Harvard Medical School, Boston, MA 02115, USA; [9]Broad Institute of MIT and Harvard, Cambridge, MA 02142, USA; [10]Department of Biomedical Informatics, Harvard Medical School, Boston, MA 02115, USA; [11]Center for Data Sciences, Brigham and Women's Hospital, Harvard Medical School, Boston, MA 02115, USA; [12]Department of Epidemiology Research, Statens Serum Institut, Copenhagen 2300, Denmark; [13]23andMe, Inc., Sunnyvale, CA 94086, USA; [14]Centre for Genetics and Genomics Versus Arthritis, Manchester Academic Health Science Centre, University of Manchester, Manchester M13 9PT, UK; [15]STACC, Tartu 51009, Estonia; [16]Department of Medicine, Vanderbilt University Medical Center, Nashville, TN 37232, USA; [17]National Institute for Health Research Oxford Biomedical Research Centre, Oxford University Hospitals NHS Foundation Trust, John Radcliffe Hospital, Oxford OX3 7LE, UK; [18]Clinical Trial Service Unit and Epidemiological Studies Unit (CTSU), Nuffield Department of Population Health, University of Oxford, Oxford OX3 7LF, UK; [19]Medical Research Council Population Health Research Unit (MRC PHRU), Nuffield Department of Population Health, University of Oxford, Oxford OX3 7LF, UK; [20]Program in Medical and Population Genetics, Broad Institute, Cambridge, MA 02142, USA; [21]Department of Pharmacology, Vanderbilt University School of Medicine, TN 37232, USA; [22]Institute for Immunology & Infectious Diseases, Murdoch University, Murdoch, WA 6150, Australia; [23]Department of Clinical Sciences, Lund University Diabetes Centre, 214 28 Malmö, Sweden; [24]Institute for Molecular Medicine Finland (FIMM), University of Helsinki, Helsinki 00014, Finland
[25]These authors contributed equally
*Correspondence: lili.milani@ut.ee
https://doi.org/10.1016/j.ajhg.2020.08.008.
© 2020 The Author(s). This is an open access article under the CC BY license (http://creativecommons.org/licenses/by/4.0/).

causes of type B reactions are antibiotics,[5] typically from the beta-lactam class, with the prevalence of penicillin allergy estimated to be as high as 25% in some settings.[7,8] Despite the relative frequency of such reactions, there are very few studies of the genetic determinants of penicillin allergy.[9,10] This underscores the need for a better understanding of the mechanisms and risk factors, including the role of genetic variation, that contribute to these reactions.

The increasing availability of genetic and phenotypic data in large biobanks provides an opportune means for investigating the role of genetic variation in drug-induced hypersensitivity reactions. In the present study, we sought to identify genetic risk factors underlying penicillin-induced hypersensitivity reactions by harnessing data from the Estonian Biobank (EstBB), UK Biobank (UKBB), and Vanderbilt University Medical Center's (VUMC) DNA Biobank (BioVU), with further replication in the 23andMe research cohort.

Subjects and Methods

Study Subjects and Phenotype Definitions

We studied individual-level genotypic and phenotypic data of 52,000 participants from the Estonian Biobank (EstBB), 500,000 participants from UK Biobank (UKBB), and a subset of 67,323 individuals from BioVU, the VUMC biorepository linked to de-identified electronic health records with self-reported European ancestry.[11] EstBB, UKBB, and BioVU are population- or hospital-based cohorts, providing a rich variety of phenotypic and health-related information collected for each participant. All participants have signed a consent form to allow follow-up linkage. In UKBB and EstBB we extracted information on penicillin allergy by searching the records of the participants for the Z88.0 ICD10 code indicating patient-reported allergy status to penicillin. Information on phenotypic features like age and gender were obtained from the biobank recruitment records. We also extracted likely penicillin allergies in EstBB from the recruitment questionnaires and free text fields of the electronic health records (EHRs) using a rule-based approach (see Supplemental Subjects and Methods for further details). In BioVU there were no records of Z88.0 diagnoses, so we used drug allergy labels from the allergy section of the EHRs, which includes adverse drug reactions reported by an individual or observed by the health care provider (Supplemental Subjects and Methods).

This study was approved by the Research Ethics Committee of the University of Tartu (Approval number 288/M-18) and conducted using the UK Biobank Resource under Application Number 11867.

Genome-wide Study and Meta-analysis

The details on genotyping, quality control, and imputation are fully described elsewhere for EstBB[12,13] and UKBB;[14] see Supplemental Subjects and Methods for further details. In EstBB, we conducted the penicillin GWAS on 44,348 individuals, including 1,320 case subjects with self-reported allergy to beta-lactam drugs or penicillin and 43,028 control subjects. In the UKBB, GWAS on penicillin allergy (defined using ICD-10 code Z88.0) was performed among 15,782 case subjects and 370,782 control subjects. In BioVU, GWAS on penicillin allergy (defined using drug allergy labels in the EHR) was performed among 12,294 case subjects and 38,284 control subjects. For all three cohorts, the GWAS was performed with SAIGE[15] including related individuals and adjusting for the first ten principal components (PCs) of the genotype matrix, as well as for age or birth year, sex (see Supplemental Subjects and Methods), and in BioVU, additionally for EHR length (years). We performed meta-analysis of 19,724,685 markers (with minor allele frequency [MAF] $> 0.1\%$) and SNP effect estimates and their standard errors were combined in a fixed effects model with the inverse variance weighted method using the METAL software.[16] Results were visualized with the R software (3.3.2) (see Web Resources).

HLA-Typing

HLA imputation of the EstBB genotype data was performed at the Broad Institute using the SNP2HLA tool.[17] The imputation was done for genotype data generated on the Global Screening Array v1, and after quality control the four-digit HLA alleles of 22,554 individuals were used for analysis. In UKBB we used four-digit imputed HLA data released by UKBB (see Web Resources).[14] The imputation process, performed using HLA*IMP:02,[18] is described fully elsewhere[14] and in the Supplemental Subjects and Methods. For the BioVU cohort, four-digit HLA-typing was imputed from SNP data with the SNP2HLA tool (Supplemental Subjects and Methods).

We performed separate additive logistic regression analysis with the called HLA alleles using R *glm* function in EstBB, UKBB, and BioVU (see Supplemental Subjects and Methods for further details). Meta-analysis of 164 HLA alleles present in all three cohorts was performed with the GWAMA software tool.[19] A Bonferroni-corrected p value threshold of 3.05×10^{-4} was applied based on the number of tested alleles (0.05/164).

For detection of the strongest tagging SNP for the HLA-B*55:01 allele, we calculated Pearson correlation coefficients between the HLA-B*55:01 allele and all the SNPs within ± 50 kb of the *HLA-B* region using the *cor* function in R (3.3.2) (see Web Resources).

HLA-B*55:01 Replication

We performed replication analysis of the HLA-B*55:01 allele in 87,996 case subjects and 1,031,087 control subjects of European ancestry (close relatives removed) from the 23andMe research cohort using an additive logistic regression model (see details in the Supplemental Subjects and Methods). The self-reported phenotype of penicillin

allergy was defined based on questionnaire data as a positive allergy test or allergic symptoms related to penicillin exposure (see Supplemental Subjects and Methods for further details). Meta-analysis of the HLA-B*55:01 association across the four cohorts was performed with the GWAMA software tool[19] and results were visualized with R software (3.3.2) (see Web Resources).

Results

Genome-wide Association Analysis of Penicillin Allergy

To discover genetic factors that may predispose to penicillin allergy, we conducted a genome-wide association study (GWAS) of 19.7 million single-nucleotide polymorphisms (SNPs) and insertions/deletions in UKBB, EstBB, and BioVu (MAF in all cohorts > 0.1%) among individuals with European ancestry. Case subjects were defined as participants with a Z88.0 ICD10 code ("Allergy status to penicillin"), which indicates a reported history of penicillin allergy (previously ICD9 "personal history of allergy to penicillin"). In total, we identified 15,782 individuals (4.1% of the total cohort size of 386,564) in UKBB with this diagnostic code. However, the corresponding number of case subjects in EstBB was only 7 (0.01% of the total cohort size of 51,936) and zero in BioVu, suggesting heterogeneity in the use of the Z88.0 ICD10 code in different countries. We therefore also identified participants that had reported drug allergy at recruitment in EstBB and categorized the EstBB self-reported reactions by drug class, using the Anatomical Therapeutic Chemical (ATC) Classification System code J01C* (beta-lactam antibacterials, penicillins) to match this to the respective Z88.0 ICD10 code. We also extracted 321 individuals with mentions of penicillin allergy in the free text fields of their EHR. This resulted in 1,320 (2.5%) case subjects with penicillin allergy in EstBB. We validated the approach in EstBB by evaluating the association between the number of filled (i.e., prescribed and purchased) penicillin (using the ATC code J01C*) prescriptions per person and self-reported penicillin allergy. Using Poisson regression analysis, we identified a negative association among individuals with self-reported allergy in EstBB on the number of filled penicillin prescriptions (p value 2.41×10^{-15}, estimate −0.18, i.e., 16% lower penicillin prescription count for individuals with penicillin allergy). In BioVU, we used drug allergy labels from the allergy section of the EHR to identify 12,294 case subjects (18.3% of the total cohort of genotyped individuals of 67,323), which is consistent with previous penicillin allergy reports using drug allergy labels.[20] To characterize the proportion of severe reactions (anaphylaxis) to penicillin among our phenotype, we analyzed the self-reported reactions among 1,017 individuals in EstBB and found that around 3% (n = 31) of the participants reported anaphylaxis and 4% (n = 42) some form of breathing difficulties. In the BioVU cohort, 5% of participants (n = 673 out of 12,294 with penicillin allergy label) reported anaphylaxis. These figures indicate that our phenotype likely captures less-severe forms of penicillin hypersensitivity.

We then meta-analyzed the results of the GWASes in these three cohorts and identified two genome-wide significant ($p < 5 \times 10^{-8}$) signals for penicillin allergy. The top hit on chromosome 6 was located in the major histocompatibility complex (MHC) region (rs114892859, MAF(EstBB) = 0.7%, MAF(UKBB) = 2%, MAF(BioVU) = 2%; p value 1.29×10^{-29}; OR 1.47 95% CI 1.38–1.57) (Figures 1A and S1, Table S1). We also identified a further signal for rs2476601, a missense variant in *PTPN22* on chromosome 1 (p value 2.68×10^{-9}; OR 1.09 95% CI 1.06–1.12).

Fine-Mapping the Penicillin Allergy-Associated HLA Locus

To further characterize the identified association with penicillin allergy, we performed a functional annotation analysis with FUMA (Functional Mapping and Annotation of Genome-Wide Association Studies).[21] We detected an independent intronic lead SNP for the penicillin allergy meta-analysis (GWAS lead variant rs114892859, p value 1.29×10^{-29}) in *MICA* (Figure 1B). When testing the SNP for expression quantitative trait locus (eQTL) associations in blood based on data from the eQTLGen Consortium,[22] the variant appeared to be associated with the expression levels of several nearby genes, with the most significant being *PSORS1C3* (p value 8.10×10^{-62}) and *MICA* (p value 1.21×10^{-52}) (Table S2). We further performed an *in silico* investigation of the lead SNP rs114892859 and its best proxy (rs144626001, the only proxy with $r^2 > 0.9$ in UKBB and EstBB) in HaploReg v.4 to explore annotations and impact of the non-coding variant.[23] rs114892859 in particular had several annotations indicative of a regulatory function, including its location in both promoter and enhancer marks in T cells and evidence of RNA polymerase II binding.[24,25] Interestingly, its proxy is more likely to be deleterious based on the scaled Combined Annotation Dependent Depletion (CADD) score (scaled score of 15.78 for rs144626001 (C/T) and 4.47 for rs114892859 (G/T)).[26,27] To assess the association of the rs114892859 variant with self-reported penicillin allergy in non-European ancestries, we used the recently developed Pan-UKB resource (see Web Resources) and retrieved summary statistics for individuals of Central/South Asian, African, East Asian, and Middle Eastern (Table S3) ancestries. We did not find an association with penicillin allergy in these other ancestry groups. Neither did we find any association of the rs114892859 variant with penicillin allergy (p value 0.288; OR 0.67 95% CI 0.14–1.19) in a subset of 14,416 BioVU individuals with self-reported African ancestry, including 1,894 case subjects and 9,539 control subjects. Nevertheless, these sample sizes are substantially smaller than the European-ancestry groups we studied and larger cohorts of diverse ancestries will be needed to provide more definitive insights.

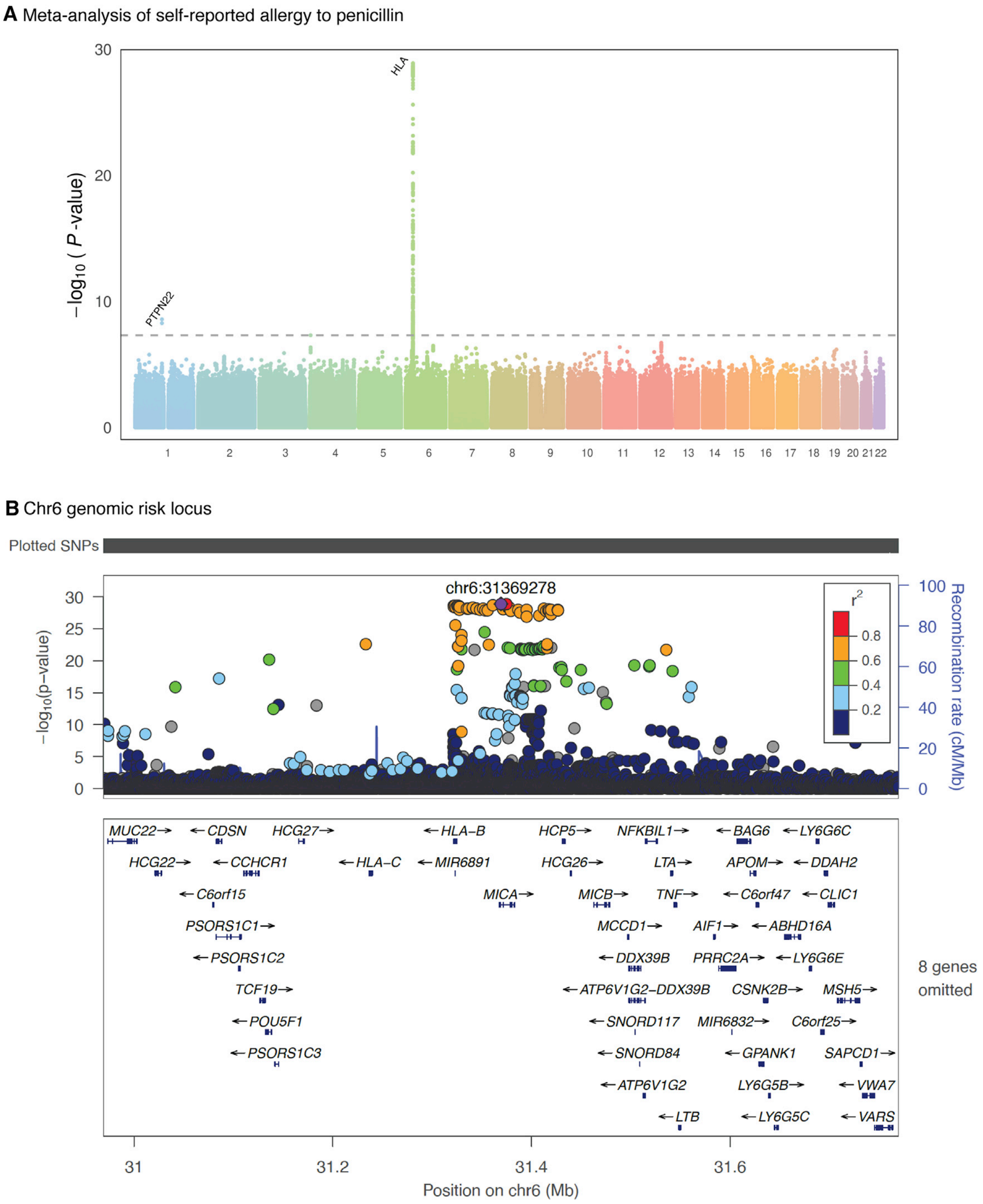

Figure 1. Manhattan Plot and HLA Locus of the Genome-wide Association Study of Penicillin Allergy

The X axes indicate chromosomal positions and Y axes $-\log_{10}$ of the p Values.

(A) Each dot represents a single-nucleotide polymorphism (SNP). The dotted line indicates the genome-wide significance (p value $< 5.0 \times 10^{-8}$) p value threshold.

(B) SNPs are colored according to their linkage disequilibrium (LD; based on the 1000 Genomes phase3 EUR reference panel) with the lead SNP. The SNP marked with a purple diamond is the lead SNP rs114892859.

Due to the high LD in the MHC region, we used imputed SNP to HLA typing data available at four-digit resolution[28] for up to 22,554, 488,377, and 67,323 individuals from the Estonian, UK, and BioVU cohorts, respectively, to further fine-map the identified HLA association with penicillin allergy. In all cohorts a shared total of 104 alleles at four-digit level were present for all of the MHC class I genes (*HLA-A, HLA-B, HLA-C*) and 60 alleles for three of the classical MHC class II genes (*HLA-DRB1, HLA-DQA1, HLA-DQB1*). To assess the variation in the frequencies of the HLA alleles in different populations, we compared the obtained allele frequencies in EstBB and UKBB (Table S4) with the

frequencies of HLA alleles in different European, Asian, and African populations reported in the HLA frequency database (Figures S2 and S3, Table S5).

We then used an additive logistic regression model to test for associations between different four-digit HLA alleles and penicillin allergy in UKBB, EstBB, and BioVU. The results from these three cohorts were meta-analyzed, using a Bonferroni-corrected p value threshold (0.05/164 = 3.05×10^{-4}, where 164 is the number of meta-analyzed HLA alleles). One of the two results that surpassed the threshold had discordant effects in the tested cohorts (Table S6). The only association with the same directional effect in all three cohorts that we detected for penicillin allergy was the HLA-B*55:01 allele (p value 2.04×10^{-31}; OR 1.41 95% CI 1.33–1.49; Table S6), which is tagged ($r^2 > 0.95$) by the GWAS lead variant rs114892859 (Table S7). We performed a separate meta-analysis for the HLA-B*55:01 allele in all case subjects from BioVU and EstBB (p value 1.98×10^{-8}; OR 1.32 95% CI 1.20–1.45) and compared it to a meta-analysis where severe reactions of anaphylaxis were excluded. Despite the smaller sample size, the estimates from this analysis were similar (p value 1.28×10^{-8}; OR 1.33 95% CI 1.20–1.46), indicating that the association is not driven by more severe hypersensitivity reactions.

Replication of the HLA-B*55:01 Association with Penicillin Allergy

To further confirm association with penicillin allergy, we analyzed the association of the HLA-B*55:01 allele with self-reported penicillin allergy among 87,996 case subjects and 1,031,087 control subjects of European ancestry from the 23andMe research cohort. We observed an association (p value 1.00×10^{-47}; OR 1.30 95% CI 1.25–1.34; Figure 2) with a similar effect size as seen for the HLA-B*55:01 allele in the meta-analysis of the EstBB, UKBB, and BioVU. Meta-analysis of estimates for HLA-B*55:01 from the discovery and replication cohorts demonstrated a 33% higher relative odds of penicillin allergy among carriers of the allele (p value 1.15×10^{-77}; OR 1.33 95% CI 1.29–1.37; Figure 2).

Further Associations at HLA-B*55:01

Finally, we used the Open Targets Genetics platform's UKBB PheWAS data[29] to further characterize the association of the GWAS lead variant (and HLA-B*55:01 allele tag-SNP) rs114892859 with other traits. We found associations with lower lymphocyte counts (p value 9.21×10^{-14}, −0.098 cells per nanoliter, per allergy-increasing T allele) and lower white blood cell counts (p value 3.17×10^{-9}, −0.078 cells per nanoliter, per allergy-increasing T allele). To confirm this finding, we extracted data on lymphocyte counts from the EHR data of 4,567 EstBB participants (see Supplemental Subjects and Methods) and observed the same inverse association of the HLA-B*55:01 allele with lymphocyte counts (−0.148 cells per nanoliter, per T allele; p value = 0.047).

To investigate the possible functional impact of the HLA-B*55:01 allele, we compared the amino acid sequence of all the *HLA-B* alleles commonly present in Estonian and UK biobank (see Supplemental Subjects and Methods). Only one allele that was represented in our populations, HLA-B*56:01, shared a high sequence similarity (>99%) with HLA-B*55:01, while all the other 46 alleles shared 86.5%–93% of the amino acids within the antigen-binding cleft (Table S8). Further analysis revealed that HLA-B*56:01 and HLA-B*55:01 differ by only two amino acids in the α2 domain: p.Glu152Val and p.Thr163Leu (Figure S4). We did not observe an association between the HLA-B*56:01 allele and penicillin allergy (p value = 0.24), which might suggest that the two amino acid differences may have functional relevance in penicillin allergy. However, despite the large number of case subjects in our study, power was limited to rule out an association with HLA-B*56:01, as it is only present at a frequency of 0.3% in European populations.

To get closer to the possible endophenotypes tagging the identified associations, we investigated genetic correlation of self-reported penicillin allergy with studies on autoimmune and hematological traits using LDhub (Table S9).[30] The analysis pointed toward genetic correlation (r_g) of 0.35 (p value 3.65×10^{-7}) between self-reported penicillin allergy and rheumatoid arthritis (RA). This result was virtually unchanged when we excluded 1 Mb around *PTPN22* ($r_g = 0.35$, p value 6.13×10^{-6}), a known RA risk locus. Since we detected this genetic correlation between RA and self-reported penicillin allergy, we redid the penicillin allergy association analysis for the HLA-B*55:01 allele among only RA case subjects in UKBB (468 penicillin allergy case subjects and 4,065 control subjects). The effect estimate for the HLA-B*55:01 allele was similar to that from the whole UKBB cohort (p value 0.032; OR 1.57 95% CI 1.04–2.38). Because LDhub did not have data for psoriasis, we further used summary statistics of the GWAS meta-analysis available for psoriasis from the PAN UKBB resource (2,868 case subjects and 417,663 control subject subjects). The genetic correlation of the GWAS meta-analysis of penicillin allergy with psoriasis was 0.44 (p value 0.002). In summary, our results suggest that the self-reported penicillin allergy phenotype could be tagging a less severe, T cell-mediated, delayed-type penicillin allergy, and that it may involve an autoimmune component.

Discussion

In the present study, we identify associations of the HLA-B*55:01 allele and a missense variant rs2476601 in *PTPN22* with self-reported penicillin allergy using data from four large cohorts: UKBB, EstBB, BioVu, and 23andMe. Hypersensitivity or allergic reactions to medications are type B adverse drug reactions that are known to be mediated by the immune system. One major driver of hypersensitivity

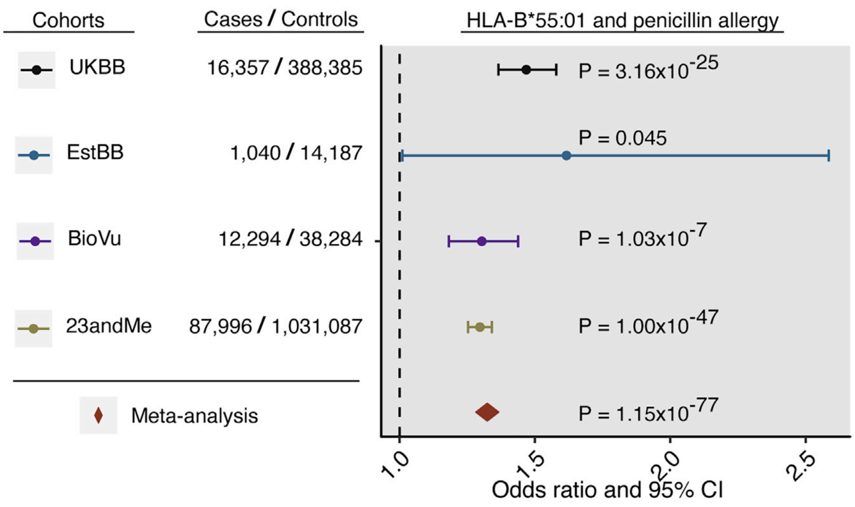

Figure 2. HLA-B*55:01 Allele Association with Self-Reported Penicillin Allergy
The odds ratios (dots) and 95% confidence intervals (CI, horizontal lines) for the association of the HLA allele with penicillin allergy are presented. The plot is annotated with p values and case-control numbers. Color coding indicates the results for discovery cohorts UKBB (black), EstBB (blue), and BioVU (purple) and replication results of the HLA-B*55:01 allele in the 23andMe research cohort (green). Results of the meta-analysis of all four cohorts is indicated with a diamond (red). Self-reported penicillin allergy is defined as ICD10 code Z88.0 (UKBB), reported drug allergy labels from the allergy section of the EHR (BioVU), reported allergy to drugs in ATC J01C* class (EstBB), or reported allergy to penicillin (23andMe).

reactions is thought to be the HLA system. HLA class I alleles are expressed on all nucleated cells. HLA is the most polymorphic region of the human genome that has played a major evolutionary role in adaptive immune responses through presentation of foreign peptides to T cell receptors that in the case of an HLA-class I restricted response leads to activation of $CD8^+$ T cells.[31] Genetic variation in the HLA region alters the shape of the peptide-binding pocket in HLA molecules and enables their binding to a vast number of different peptides—a crucial step in the adaptive immune response.[32] However, this ability of HLA molecules to bind a wide variety of peptides may also facilitate binding of exogenous molecules such as drugs, potentially leading to off-target drug effects and immune-mediated ADRs.[33] The precise mechanism of most HLA-drug interactions remains unknown, but it seems that T cell activation is necessary for the majority of HLA-mediated ADRs.[33–35] Despite the increasing evidence for a role of the HLA system in drug-induced hypersensitivity, much is still unclear mechanistically as to how genetic variation in the HLA region predisposes to specific drug reactions.

Penicillin is the most common cause of drug allergy, with clinical manifestations ranging from relatively benign cutaneous reactions to life-threatening systemic syndromes.[7,8] There is a previous GWAS on the immediate type of penicillin allergy, where a borderline genome-wide significant protective association of an allele of the MHC class II gene *HLA-DRA* was detected and further replicated in a different cohort.[36] Here we detect a robust association between self-reported penicillin allergy and an allele of the MHC class I gene *HLA-B*. The allele and its tag-SNP were also associated with lower lymphocyte counts and overlapped with T cell regulatory annotations. This raises the possibility that the variant may predispose to a T cell-mediated process that could lead to a delayed penicillin reaction through a heterologous response from an HLA-B*55:01 restricted immune response that occurred earlier in life to a prevalent pathogen or an infection or disease interaction. MHC I molecules are expressed by almost all cells and present peptides to cytotoxic $CD8^+$ T cells, whereas MHC II molecules are expressed by antigen-presenting cells to present peptides to $CD4^+$ T helper lymphocyte.[32,35] There are several examples of MHC I alleles associated with drug-induced hypersensitivity mediated by $CD8^+$ T cells.[35,37,38] The involvement of T cells in delayed hypersensitivity reactions has been shown by isolating drug-reactive T cell clones,[39] and cytotoxic $CD8^+$ T cells have been shown to be relevant especially in allergic skin reactions.[40–42] More than 20 years ago, $CD8^+$ T cells reactive to penicillin were isolated from patients with delayed type of hypersensitivity to penicillin.[43] The association with the HLA-B*55:01 allele detected in our study might be a relevant factor in this established connection with $CD8^+$ T cells as HLA-B07-supertype alleles that share peptide binding specificities with HLA-B*55:01 have previously been associated with nevirapine-induced rash.[44] The underlying mechanism in penicillin allergy remains a question and various models have been proposed for T cell-mediated hypersensitivity.[37,42] For example, the hapten model suggests that drugs may alter proteins and thereby induce an immune response[37,45]—penicillins have been shown to bind proteins[45,46] to form hapten-carrier complexes, which may in turn elicit a T cell response.[47] Drugs may also non-covalently interact with MHC molecules and alter the repertoire of bound peptides leading to presentation of antigens to which the host has not been previously tolerized. For example, abacavir has been shown to bind non-covalently within the F pocket of the antigen binding cleft of HLA-B*57:01, altering its peptide specificity and leading to a $CD8^+$ T cell-mediated hypersensitivity response.[48–50]

It is increasingly recognized that the involvement of HLA variation in hypersensitivity reactions goes beyond peptide specificity. Other factors, such as effects on HLA expression that influence the strength of the immune response, have also been described.[51] The analysis of eQTLs based on the data of the eQTLGen Consortium[22] revealed that the lead SNP rs114892859 identified in our GWAS of penicillin allergy appears to be associated with

the expression of several nearby genes, including expression of both *HLA-B* and *HLA-C*, and an even stronger effect on RNA levels of *PSORS1C3* and *MICA* (Table S2). Variants in *PSORS1C3* have been associated with the risk of allopurinol-, carbamazepine-, and phenytoin-induced SJS/TEN hypersensitivity reactions[52] and *MICA* encodes the protein MHC class I polypeptide-related sequence A[53] which has been implicated in immune surveillance.[54,55] Our findings therefore support the observation that variants associated with expression of HLA genes may contribute to the development of hypersensitivity reactions.

We also detected an association with variants in *PTPN22* on chromosome 1. *PTPN22* encodes a tyrosine phosphatase involved in the regulation of immune cell signaling.[56] The lead missense variant rs2476601 has previously been associated with several autoimmune diseases[57] and is a risk allele for rheumatoid arthritis.[58] Interestingly, this variant was also recently shown to be associated with drug-induced liver injury (DILI).[59] The association with the rs2476601 variant was strongest for cases of amoxicillin- and clavulanic acid-associated liver injury (OR 1.62, p value 4.0×10^{-6}). Case subjects in this study were clinically sourced and comprehensively phenotyped, which suggests that our self-reported penicillin allergy phenotype might also capture signal related to more severe forms of beta-lactam hypersensitivity. However, the effect of this variant on penicillin allergy in the current study is relatively small (OR 1.09) and its role in the development of allergic reactions needs further studies.

A genetic correlation analysis of the penicillin allergy GWAS results in the current study revealed overlap with rheumatoid arthritis, even when excluding the *PTPN22* region from the analysis. Furthermore, we identified a genetic correlation with psoriasis, another autoimmune disease. Both psoriasis and psoriatic arthritis also have associations with *HLA-B* alleles.[60,61] This indicates a possible underlying autoimmune factor in the development of the penicillin allergy phenotype investigated in our study.

Studies have suggested that penicillin allergy labels are acquired in childhood and of children labeled as penicillin allergic, 75% have acquired this label by age 3.[8] In addition, approximately 10% of patients tested per year will lose their skin test reactivity, meaning that by adulthood >95% of allergy labels can be removed with formal testing. The main limitation of this study is the unverified nature of the phenotypes extracted from EHRs and self-reported data in the biobanks. Previous work has found that most (90%–95%) individuals labeled as having beta-lactam hypersensitivity may not actually have true hypersensitivity by adulthood when they more commonly undergo validated testing.[7,8,62,63] However, we believe that the phenotype we have studied is valid for several reasons.[7,62] The most commonly reported penicillin allergy is delayed-type allergy, which usually manifests as a transient benign rash that does not recur on rechallenge many years later.[7,62,64] Furthermore, many individuals who were once labeled as having IgE-mediated penicillin allergy develop tolerance over time.[7] In both of these cases, a true penicillin-induced reaction occurred initially, even if tolerance develops subsequently. Our phenotype therefore could represent individuals that experienced a reaction associated to penicillin when they were previously exposed, but who may, over time, tolerate penicillin administration. A delayed rash is the most common self-reported reaction seen in association with penicillin,[7,62,64] which is frequently a T cell-mediated process. The results from our *in silico* analyses, which link HLA-B*55:01 to T cell biology, would support this observation. Therefore, we posit that the association with the HLA-B*55:01 allele may represent a predisposition to an immune response associated with penicillin which does not appear to be associated with a severe immediate or delayed reaction and which may wane with time.

Despite the possibility that some cases in our study may be misclassified, we detect a robust HLA association that was replicated in several independent cohorts against related phenotypes. The increased power arising from biobank-scale sample sizes therefore mitigates some of the challenges associated with EHR data. The robustness of the genetic signal across cohorts with orthogonal phenotyping methods, ranging from EHR-sourced in EstBB and BioVU to various forms of self-reported data in UKBB and 23andMe, also supports a true association. Finally, the modest effect size of the HLA-B*55:01 allele (OR 1.33), particularly when compared to effect sizes of HLA alleles with established pharmacogenetic relevance,[65–67] suggests that this variant has limited predictive value. However, further phenotypic refinement, including investigation of specific penicillin derivatives and specific types of drug reactions, may yield more clinically actionable insight.

In summary, we have leveraged data from four large-scale cohorts, including more than 100,000 case subjects, to provide insights into the genetic architecture of self-reported penicillin allergy and to provide robust evidence implicating the HLA-B*55:01 allele in this condition. Further studies are necessary to determine the precise underlying immune processes and how these change over time.

Data and Code Availability

All code used for the analysis is described in the Subjects and Methods section of the manuscript. Genotype and phenotype data are available from the Estonian Biobank (https://genomics.ut.ee/en/biobank.ee/data-access) and UK Biobank (https://www.ukbiobank.ac.uk/using-the-resource/) upon request.

Supplemental Data

Supplemental Data can be found online at https://doi.org/10.1016/j.ajhg.2020.08.008.

Consortia

The members of 23andMe research team: Michelle Agee, Stella Aslibekyan, Robert K. Bell, Katarzyna Bryc, Sarah K. Clark, Sarah L. Elson, Kipper Fletez-Brant, Pierre Fontanillas, Nicholas A. Furlotte, Pooja M. Gandhi, Karl Heilbron, Barry Hicks, David A. Hinds, Karen E. Huber, Ethan M. Jewett, Yunxuan Jiang, Aaron Kleinman, Keng-Han Lin, Nadia K. Litterman, Marie K. Luff, Jennifer C. McCreight, Matthew H. McIntyre, Kimberly F. McManus, Joanna L. Mountain, Sahar V. Mozaffari, Priyanka Nandakumar, Elizabeth S. Noblin, Carrie A.M. Northover, Jared O'Connell, Aaron A. Petrakovitz, Steven J. Pitts, G. David Poznik, J. Fah Sathirapongsasuti, Anjali J. Shastri, Janie F. Shelton, Suyash Shringarpure, Chao Tian, Joyce Y. Tung, Robert J. Tunney, Vladimir Vacic, Xin Wang, and Amir S. Zare.

Author Contributions

K.K., L.M., and J.F. designed the study. R.M., M.L., Y.L., S.R., E.J.P., D.M.R., W.-Q.W., A.M., and T.E. supervised and generated genotype data or HLA typing data. D.S. and S.L. generated allergy data from free text. K.K., J.B., N.Z., M.L., T.J., J.C.C., T.L., J.F., W.W., and A.A. performed the data analysis. E.A. conducted amino acid sequence analysis. K.K., J.B., N.Z., M.V.H., C.M.L., R.M., L.M., J.C.C., E.J.P., W.-Q.W., and J.F. conducted data interpretation. K.K. prepared the figures and tables. K.K., J.B., L.M., and J.F. drafted the manuscript. K.K., J.B., N.Z., M.V.H., C.M.L., M.L., R.M., L.M., J.C.C., W.W., A.A., E.J.P., J.F., B.F., F.G., and L.S. reviewed and edited the manuscript. All authors contributed to critical revisions and approved the final manuscript.

The corresponding author attests that all listed authors meet authorship criteria and that no others meeting the criteria have been omitted.

Acknowledgments

We thank all participants and staff of the Estonian, UK, and BioVU biobanks, and 23andMe for their contribution to this research. This work was carried out in part in the High Performance Computing Center of the University of Tartu.

This study has been supported by grants from the Estonian Research Council (PRG184, PRG687, IUT20-60, and IUT24-6) and the Oak Foundation. The BioVU analyses used Vanderbilt University Medical Center's resources, the Synthetic Derivative, which are supported by institutional funding and the National Center for Advancing Translational Science grant 2UL1 TR000445-06. J.B. is supported by the Rhodes Trust, Clarendon Fund, and the Medical Sciences Doctoral Training Centre, University of Oxford. J.C.C. is funded by the Oxford Medical Research Council Doctoral Training Partnership and the Nuffield Department of Clinical Medicine, University of Oxford. C.M.L. is supported by the Li Ka Shing Foundation; WT-SSI/John Fell funds; the NIHR Biomedical Research Centre, Oxford; Widenlife; and NIH (5P50HD028138-27). M.V.H. is supported by a BHF Intermediate Clinical Research Fellowship (FS/18/23/33512) and the NIHR Biomedical Research Centre, Oxford. Computation used the Oxford Biomedical Research Computing (BMRC) facility, a joint development between the Wellcome Centre for Human Genetics and the Big Data Institute supported by Health Data Research UK and the NIHR Oxford Biomedical Research Centre. Financial support was provided by the Wellcome Trust Core Award Grant Number 203141/Z/16/Z (see full acknowledgments in the Supplemental Information). E.J.P. receives support from NIH (P50GM115305 [also D.M.R. and W.-Q.W.], R01HG010863, R01AI152183, R21AI139021, U01AI154659) and the National Health and Medical Research Council of Australia.

Declaration of Interests

C.M.L. has collaborated with Novo Nordisk and Bayer in research, and in accordance with a university agreement, did not accept any personal payment. W.W., A.A., and members of the 23andMe Research Team are employed by and hold stock or stock options in 23andMe, Inc. There were no other relationships or activities that could appear to have influenced the submitted work. The views expressed are those of the author(s) and not necessarily those of the NHS, the NIHR, the Department of Health, or the NIH.

Received: April 17, 2020
Accepted: August 10, 2020
Published: September 3, 2020

Web Resources

Pan-UKB, https://pan.ukbb.broadinstitute.org
R statistical software, https://www.r-project.org/
UK Biobank: Resource 182, https://biobank.ctsu.ox.ac.uk/crystal/refer.cgi?id=182

References

1. Lazarou, J., Pomeranz, B.H., and Corey, P.N. (1998). Incidence of adverse drug reactions in hospitalized patients: a meta-analysis of prospective studies. JAMA *279*, 1200–1205.
2. Santoro, A., Genov, G., Spooner, A., Raine, J., and Arlett, P. (2017). Promoting and Protecting Public Health: How the European Union Pharmacovigilance System Works. Drug Saf. *40*, 855–869.
3. Bouvy, J.C., De Bruin, M.L., and Koopmanschap, M.A. (2015). Epidemiology of adverse drug reactions in Europe: a review of recent observational studies. Drug Saf. *38*, 437–453.
4. Alagoz, O., Durham, D., and Kasirajan, K. (2016). Cost-effectiveness of one-time genetic testing to minimize lifetime adverse drug reactions. Pharmacogenomics J. *16*, 129–136.
5. Böhm, R., and Cascorbi, I. (2016). Pharmacogenetics and predictive testing of drug hypersensitivity reactions. Front. Pharmacol. *7*, 396.
6. Iasella, C.J., Johnson, H.J., and Dunn, M.A. (2017). Adverse Drug Reactions: Type A (Intrinsic) or Type B (Idiosyncratic). Clin. Liver Dis. *21*, 73–87.
7. Blumenthal, K.G., Peter, J.G., Trubiano, J.A., and Phillips, E.J. (2019). Antibiotic allergy. Lancet *393*, 183–198.
8. Castells, M., Khan, D.A., and Phillips, E.J. (2019). Penicillin Allergy. N. Engl. J. Med. *381*, 2338–2351.
9. Mirakian, R., Leech, S.C., Krishna, M.T., Richter, A.G., Huber, P.A.J., Farooque, S., Khan, N., Pirmohamed, M., Clark, A.T., Nasser, S.M.; and Standards of Care Committee of the British Society for Allergy and Clinical Immunology (2015). Management of allergy to penicillins and other beta-lactams. Clin. Exp. Allergy *45*, 300–327.
10. Drug and Therapeutics Bulletin (2017). Penicillin allergy-getting the label right. BMJ *358*, j3402.

11. Roden, D.M., Pulley, J.M., Basford, M.A., Bernard, G.R., Clayton, E.W., Balser, J.R., and Masys, D.R. (2008). Development of a large-scale de-identified DNA biobank to enable personalized medicine. Clin. Pharmacol. Ther. *84*, 362–369.
12. Mitt, M., Kals, M., Pärn, K., Gabriel, S.B., Lander, E.S., Palotie, A., Ripatti, S., Morris, A.P., Metspalu, A., Esko, T., et al. (2017). Improved imputation accuracy of rare and low-frequency variants using population-specific high-coverage WGS-based imputation reference panel. Eur. J. Hum. Genet. *25*, 869–876.
13. Kals, M., Nikopensius, T., Läll, K., Pärn, K., Sikka, T.T., Suvisaari, J., Salomaa, V., Ripatti, S., Palotie, A., Metspalu, A., et al. (2019). Advantages of genotype imputation with ethnically matched reference panel for rare variant association analyses. bioRxiv. https://doi.org/10.1101/579201.
14. Bycroft, C., Freeman, C., Petkova, D., Band, G., Elliott, L.T., Sharp, K., Motyer, A., Vukcevic, D., Delaneau, O., O'Connell, J., et al. (2018). The UK Biobank resource with deep phenotyping and genomic data. Nature *562*, 203–209.
15. Zhou, W., Nielsen, J.B., Fritsche, L.G., Dey, R., Gabrielsen, M.E., Wolford, B.N., LeFaive, J., VandeHaar, P., Gagliano, S.A., Gifford, A., et al. (2018). Efficiently controlling for case-control imbalance and sample relatedness in large-scale genetic association studies. Nat. Genet. *50*, 1335–1341.
16. Willer, C.J., Li, Y., and Abecasis, G.R. (2010). METAL: fast and efficient meta-analysis of genomewide association scans. Bioinformatics *26*, 2190–2191.
17. Jia, X., Han, B., Onengut-Gumuscu, S., Chen, W.-M., Concannon, P.J., Rich, S.S., Raychaudhuri, S., and de Bakker, P.I.W. (2013). Imputing amino acid polymorphisms in human leukocyte antigens. PLoS ONE *8*, e64683.
18. Dilthey, A., Leslie, S., Moutsianas, L., Shen, J., Cox, C., Nelson, M.R., and McVean, G. (2013). Multi-population classical HLA type imputation. PLoS Comput. Biol. *9*, e1002877.
19. Mägi, R., and Morris, A.P. (2010). GWAMA: software for genome-wide association meta-analysis. BMC Bioinformatics *11*, 288.
20. Zhou, L., Dhopeshwarkar, N., Blumenthal, K.G., Goss, F., Topaz, M., Slight, S.P., and Bates, D.W. (2016). Drug allergies documented in electronic health records of a large healthcare system. Allergy *71*, 1305–1313.
21. Watanabe, K., Taskesen, E., van Bochoven, A., and Posthuma, D. (2017). Functional mapping and annotation of genetic associations with FUMA. Nat. Commun. *8*, 1826.
22. Võsa, U., Claringbould, A., Westra, H.-J., Bonder, M.J., Deelen, P., Zeng, B., Kirsten, H., Saha, A., Kreuzhuber, R., Kasela, S., et al. (2018). Unraveling the polygenic architecture of complex traits using blood eQTL metaanalysis. bioRxiv. https://doi.org/10.1101/447367.
23. Ward, L.D., and Kellis, M. (2012). HaploReg: a resource for exploring chromatin states, conservation, and regulatory motif alterations within sets of genetically linked variants. Nucleic Acids Res. *40*, D930–D934.
24. Kundaje, A., Meuleman, W., Ernst, J., Bilenky, M., Yen, A., Heravi-Moussavi, A., Kheradpour, P., Zhang, Z., Wang, J., Ziller, M.J., et al.; Roadmap Epigenomics Consortium (2015). Integrative analysis of 111 reference human epigenomes. Nature *518*, 317–330.
25. Myers, R.M., Stamatoyannopoulos, J., Snyder, M., Dunham, I., Hardison, R.C., Bernstein, B.E., Gingeras, T.R., Kent, W.J., Birney, E., Wold, B., et al.; ENCODE Project Consortium (2011). A user's guide to the encyclopedia of DNA elements (ENCODE). PLoS Biol. *9*, e1001046.
26. Rentzsch, P., Witten, D., Cooper, G.M., Shendure, J., and Kircher, M. (2019). CADD: predicting the deleteriousness of variants throughout the human genome. Nucleic Acids Res. *47* (D1), D886–D894.
27. Kircher, M., Witten, D.M., Jain, P., O'Roak, B.J., Cooper, G.M., and Shendure, J. (2014). A general framework for estimating the relative pathogenicity of human genetic variants. Nat. Genet. *46*, 310–315.
28. Marsh, S.G.E., Albert, E.D., Bodmer, W.F., Bontrop, R.E., Dupont, B., Erlich, H.A., Fernández-Viña, M., Geraghty, D.E., Holdsworth, R., Hurley, C.K., et al. (2010). Nomenclature for factors of the HLA system, 2010. Tissue Antigens *75*, 291–455.
29. Koscielny, G., An, P., Carvalho-Silva, D., Cham, J.A., Fumis, L., Gasparyan, R., Hasan, S., Karamanis, N., Maguire, M., Papa, E., et al. (2017). Open Targets: a platform for therapeutic target identification and validation. Nucleic Acids Res. *45* (D1), D985–D994.
30. Zheng, J., Erzurumluoglu, A.M., Elsworth, B.L., Kemp, J.P., Howe, L., Haycock, P.C., Hemani, G., Tansey, K., Laurin, C., Pourcain, B.S., et al.; Early Genetics and Lifecourse Epidemiology (EAGLE) Eczema Consortium (2017). LD Hub: a centralized database and web interface to perform LD score regression that maximizes the potential of summary level GWAS data for SNP heritability and genetic correlation analysis. Bioinformatics *33*, 272–279.
31. Williams, T.M. (2001). Human leukocyte antigen gene polymorphism and the histocompatibility laboratory. J. Mol. Diagn. *3*, 98–104.
32. Chaplin, D.D. (2010). Overview of the immune response. J. Allergy Clin. Immunol. *125* (2, Suppl 2), S3–S23.
33. Illing, P.T., Purcell, A.W., and McCluskey, J. (2017). The role of HLA genes in pharmacogenomics: unravelling HLA associated adverse drug reactions. Immunogenetics *69*, 617–630.
34. Pavlos, R., Mallal, S., and Phillips, E. (2012). HLA and pharmacogenetics of drug hypersensitivity. Pharmacogenomics *13*, 1285–1306.
35. Negrini, S., and Becquemont, L. (2017). HLA-associated drug hypersensitivity and the prediction of adverse drug reactions. Pharmacogenomics *18*, 1441–1457.
36. Guéant, J.L., Romano, A., Cornejo-Garcia, J.A., Oussalah, A., Chery, C., Blanca-López, N., Guéant-Rodriguez, R.M., Gaeta, F., Rouyer, P., Josse, T., et al. (2015). HLA-DRA variants predict penicillin allergy in genome-wide fine-mapping genotyping. J. Allergy Clin. Immunol. *135*, 253–259.
37. Pavlos, R., Mallal, S., Ostrov, D., Buus, S., Metushi, I., Peters, B., and Phillips, E. (2015). T cell-mediated hypersensitivity reactions to drugs. Annu. Rev. Med. *66*, 439–454.
38. Sousa-Pinto, B., Correia, C., Gomes, L., Gil-Mata, S., Araújo, L., Correia, O., and Delgado, L. (2016). HLA and delayed drug-induced hypersensitivity. Int. Arch. Allergy Immunol. *170*, 163–179.
39. Yawalkar, N., Egli, F., Hari, Y., Nievergelt, H., Braathen, L.R., and Pichler, W.J. (2000). Infiltration of cytotoxic T cells in drug-induced cutaneous eruptions. Clin. Exp. Allergy *30*, 847–855.
40. Kalish, R.S., and Askenase, P.W. (1999). Molecular mechanisms of CD8+ T cell-mediated delayed hypersensitivity: implications for allergies, asthma, and autoimmunity. J. Allergy Clin. Immunol. *103*, 192–199.

41. Romano, A., Blanca, M., Torres, M.J., Bircher, A., Aberer, W., Brockow, K., Pichler, W.J., Demoly, P.; ENDA; and EAACI (2004). Diagnosis of nonimmediate reactions to beta-lactam antibiotics. Allergy *59*, 1153–1160.
42. Adam, J., Pichler, W.J., and Yerly, D. (2011). Delayed drug hypersensitivity: models of T-cell stimulation. Br. J. Clin. Pharmacol. *71*, 701–707.
43. Hertl, M., Geisel, J., Boecker, C., and Merk, H.F. (1993). Selective generation of CD8+ T-cell clones from the peripheral blood of patients with cutaneous reactions to beta-lactam antibiotics. Br. J. Dermatol. *128*, 619–626.
44. Pavlos, R., McKinnon, E.J., Ostrov, D.A., Peters, B., Buus, S., Koelle, D., Chopra, A., Schutte, R., Rive, C., Redwood, A., et al. (2017). Shared peptide binding of HLA Class I and II alleles associate with cutaneous nevirapine hypersensitivity and identify novel risk alleles. Sci. Rep. *7*, 8653.
45. Pirmohamed, M., Ostrov, D.A., and Park, B.K. (2015). New genetic findings lead the way to a better understanding of fundamental mechanisms of drug hypersensitivity. J. Allergy Clin. Immunol. *136*, 236–244.
46. Meng, X., Jenkins, R.E., Berry, N.G., Maggs, J.L., Farrell, J., Lane, C.S., Stachulski, A.V., French, N.S., Naisbitt, D.J., Pirmohamed, M., and Park, B.K. (2011). Direct evidence for the formation of diastereoisomeric benzylpenicilloyl haptens from benzylpenicillin and benzylpenicillenic acid in patients. J. Pharmacol. Exp. Ther. *338*, 841–849.
47. Weltzien, H.U., and Padovan, E. (1998). Molecular features of penicillin allergy. J. Invest. Dermatol. *110*, 203–206.
48. Chessman, D., Kostenko, L., Lethborg, T., Purcell, A.W., Williamson, N.A., Chen, Z., Kjer-Nielsen, L., Mifsud, N.A., Tait, B.D., Holdsworth, R., et al. (2008). Human leukocyte antigen class I-restricted activation of CD8+ T cells provides the immunogenetic basis of a systemic drug hypersensitivity. Immunity *28*, 822–832.
49. Ostrov, D.A., Grant, B.J., Pompeu, Y.A., Sidney, J., Harndahl, M., Southwood, S., Oseroff, C., Lu, S., Jakoncic, J., de Oliveira, C.A.F., et al. (2012). Drug hypersensitivity caused by alteration of the MHC-presented self-peptide repertoire. Proc. Natl. Acad. Sci. USA *109*, 9959–9964.
50. Illing, P.T., Vivian, J.P., Dudek, N.L., Kostenko, L., Chen, Z., Bharadwaj, M., Miles, J.J., Kjer-Nielsen, L., Gras, S., Williamson, N.A., et al. (2012). Immune self-reactivity triggered by drug-modified HLA-peptide repertoire. Nature *486*, 554–558.
51. Aguiar, V.R.C., César, J., Delaneau, O., Dermitzakis, E.T., and Meyer, D. (2019). Expression estimation and eQTL mapping for HLA genes with a personalized pipeline. PLoS Genet. *15*, e1008091.
52. Génin, E., Schumacher, M., Roujeau, J.C., Naldi, L., Liss, Y., Kazma, R., Sekula, P., Hovnanian, A., and Mockenhaupt, M. (2011). Genome-wide association study of Stevens-Johnson Syndrome and Toxic Epidermal Necrolysis in Europe. Orphanet J. Rare Dis. *6*, 52.
53. Bateman, A.; and UniProt Consortium (2019). UniProt: a worldwide hub of protein knowledge. Nucleic Acids Res. *47* (D1), D506–D515.
54. Duan, Q., Li, H., Gao, C., Zhao, H., Wu, S., Wu, H., Wang, C., Shen, Q., and Yin, T. (2019). High glucose promotes pancreatic cancer cells to escape from immune surveillance via AMPK-Bmi1-GATA2-MICA/B pathway. J. Exp. Clin. Cancer Res. *38*, 192.
55. Shafi, S., Vantourout, P., Wallace, G., Antoun, A., Vaughan, R., Stanford, M., and Hayday, A. (2011). An NKG2D-mediated human lymphoid stress surveillance response with high interindividual variation. Sci. Transl. Med. *3*, 113ra124.
56. Stanford, S.M., Rapini, N., and Bottini, N. (2012). Regulation of TCR signalling by tyrosine phosphatases: from immune homeostasis to autoimmunity. Immunology *137*, 1–19.
57. Stanford, S.M., and Bottini, N. (2014). PTPN22: the archetypal non-HLA autoimmunity gene. Nat. Rev. Rheumatol. *10*, 602–611.
58. Begovich, A.B., Carlton, V.E.H., Honigberg, L.A., Schrodi, S.J., Chokkalingam, A.P., Alexander, H.C., Ardlie, K.G., Huang, Q., Smith, A.M., Spoerke, J.M., et al. (2004). A missense single-nucleotide polymorphism in a gene encoding a protein tyrosine phosphatase (PTPN22) is associated with rheumatoid arthritis. Am. J. Hum. Genet. *75*, 330–337.
59. Cirulli, E.T., Nicoletti, P., Abramson, K., Andrade, R.J., Bjornsson, E.S., Chalasani, N., Fontana, R.J., Hallberg, P., Li, Y.J., Lucena, M.I., et al.; Drug-Induced Liver Injury Network (DILIN) investigators; and International DILI consortium (iDILIC) (2019). A Missense Variant in PTPN22 is a Risk Factor for Drug-induced Liver Injury. Gastroenterology *156*, 1707–1716.e2.
60. Chen, H., Hayashi, G., Lai, O.Y., Dilthey, A., Kuebler, P.J., Wong, T.V., Martin, M.P., Fernandez Vina, M.A., McVean, G., Wabl, M., et al. (2012). Psoriasis patients are enriched for genetic variants that protect against HIV-1 disease. PLoS Genet. *8*, e1002514.
61. Winchester, R., Giles, J., Jadon, D., Haroon, M., McHugh, N., and FitzGerald, O. (2016). Implications of the diversity of class I HLA associations in psoriatic arthritis. Clin. Immunol. *172*, 29–33.
62. Shenoy, E.S., Macy, E., Rowe, T., and Blumenthal, K.G. (2019). Evaluation and Management of Penicillin Allergy: A Review. JAMA *321*, 188–199.
63. Jani, Y.H., Williams, I., and Krishna, M.T. (2020). Sustaining and spreading penicillin allergy delabelling: A narrative review of the challenges for service delivery and patient safety. Br. J. Clin. Pharmacol. *86*, 548–559.
64. Sousa-Pinto, B., Fonseca, J.A., and Gomes, E.R. (2017). Frequency of self-reported drug allergy: A systematic review and meta-analysis with meta-regression. Ann. Allergy Asthma Immunol. *119*, 362–373.e2.
65. Mallal, S., Nolan, D., Witt, C., Masel, G., Martin, A.M., Moore, C., Sayer, D., Castley, A., Mamotte, C., Maxwell, D., et al. (2002). Association between presence of HLA-B*5701, HLA-DR7, and HLA-DQ3 and hypersensitivity to HIV-1 reverse-transcriptase inhibitor abacavir. Lancet *359*, 727–732.
66. Chen, P., Lin, J.-J., Lu, C.-S., Ong, C.-T., Hsieh, P.F., Yang, C.-C., Tai, C.-T., Wu, S.-L., Lu, C.-H., Hsu, Y.-C., et al.; Taiwan SJS Consortium (2011). Carbamazepine-induced toxic effects and HLA-B*1502 screening in Taiwan. N. Engl. J. Med. *364*, 1126–1133.
67. McCormack, M., Alfirevic, A., Bourgeois, S., Farrell, J.J., Kasperavičiūtė, D., Carrington, M., Sills, G.J., Marson, T., Jia, X., de Bakker, P.I.W., et al. (2011). HLA-A*3101 and carbamazepine-induced hypersensitivity reactions in Europeans. N. Engl. J. Med. *364*, 1134–1143.

The American Journal of Human Genetics, Volume *107*

Supplemental Data

Genome-wide Study Identifies Association between HLA-B*55:01 and Self-Reported Penicillin Allergy

Kristi Krebs, Jonas Bovijn, Neil Zheng, Maarja Lepamets, Jenny C. Censin, Tuuli Jürgenson, Dage Särg, Erik Abner, Triin Laisk, Yang Luo, Line Skotte, Frank Geller, Bjarke Feenstra, Wei Wang, Adam Auton, 23andMe Research Team, Soumya Raychaudhuri, Tõnu Esko, Andres Metspalu, Sven Laur, Dan M. Roden, Wei-Qi Wei, Michael V. Holmes, Cecilia M. Lindgren, Elizabeth J. Phillips, Reedik Mägi, Lili Milani, and João Fadista

Supplemental Data

Supplemental Figures *3*

Figure S1. The quantile-quantile (QQ) plot for the genome-wide meta-analysis of self-reported penicillin allergy. The observed lambda value is 1.03. 3

Figure S2. The distribution of allele frequencies of MHC class I genes HLA-A, HLA-B and HLA-C in European (darker grey), Asian (lighter grey) and African (green) populations. The frequency of allels in Estonian (blue) and UK (red) biobanks are shown in respectively colored dots. 4

Figure S3. The distribution of allele frequencies of MHC class II genes HLA-DRB1, HLA-DQB1 and HLA-DQA1 in European (darker grey), Asian (lighter grey) and African (green) populations. The frequency of allels in Estonian (blue) and UK (red) biobanks are shown in respectively colored dots. 5

Figure S4. HLA-B*55:01 exhibits structural differences from another common HLA-B allele. Yellow residues highlight the two different amino acids in the antigen-binding cleft between 55:01 (left) and 56:01 (right). 6

Supplemental Tables *7*

Table S1. Genome-wide significant associations of meta-analysis of penicillin allergy 7

Table S2. Associations of expression quantitative trait locus (eQTL) in blood with the results of penicillin allergy meta-analysis based on the eQTLGen Consortium data. eQTLGen is a meta-analysis of cis-/trans-eQTLs from 37 datasets with a total of 31,684 individuals. Signed stats column indicates the direction that is either "+" indicating that risk increasing allele increases the expression of the gene or "-" indicating the risk increasing allele decreases the expression of the gene. 7

Table S3. Summary statistics of association of the rs114892859 variant with penicillin allergy in non-European ancestries based on the Pan-UKB database 8

Table S4. The frequencies of HLA four-digit alleles in Estonian and UK biobank. 9

Table S5. The frequency difference test between European vs Asian and European vs African populations using Wilcoxon test for all HLA alleles. 9

Table S6. Summary statistics of associations between penicillin allergy and four-digit HLA haplotypes in Estonian, UK and BioVU biobank. 9

Table S7. The HLA-B*5501 allele correlation with the SNPs in the HLA region in Estonian and UK biobank. 10

Table S8. Amino acid sequence similarity of 48 HLA-B allele serotypes present in Estonian and UK biobanks. Cells Green and yellow colouring indicates the similarity between values within columns (%), while blue and red colouring indicate a binary amino acid conservation within the specific column. 11

Table S9. Genetic correlation of self-reported penicillin allergy with published autoimmune and hematological traits using LDHub. PMID – PubMed ID (reference study); rg- genetic correlation; se- standard error of rg; z- z-score of rg; p- p-value of rg. 12

Supplemental Methods *13*

Phenotype definitions 13

Genotype information 14
Genome-wide study of penicillin allergy 16
Post-GWAS annotation 16
HLA typing 17
Comparison of HLA allele frequencies 18
Replication in 23andMe 19
HLA-B*55:01 allele association with lymphocyte levels in EstBB 19
Comparison of the amino acid sequences of HLA-B alleles 20
Supplemental Acknowledgements *20*
Supplemental References *21*

Supplemental Figures

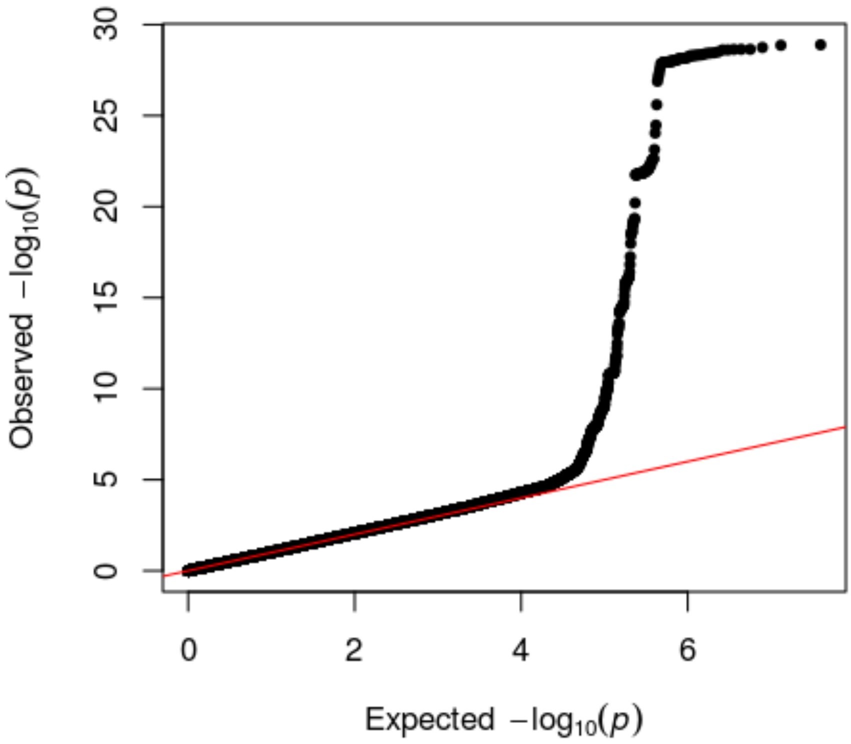

Figure S1. The quantile-quantile (QQ) plot for the genome-wide meta-analysis of self-reported penicillin allergy. The observed lambda value is 1.03.

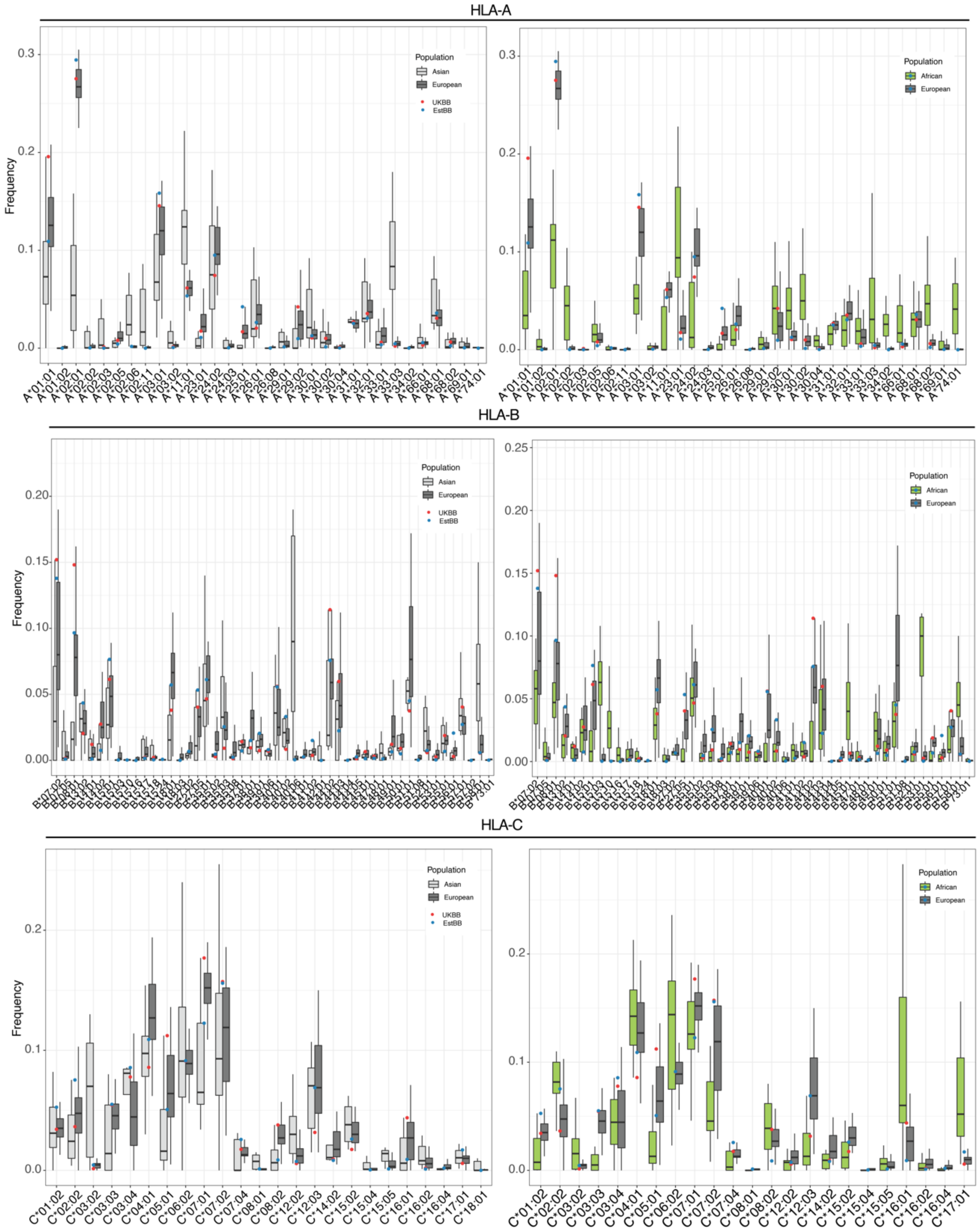

Figure S2. The distribution of allele frequencies of MHC class I genes HLA-A, HLA-B and HLA-C in European (darker grey), Asian (lighter grey) and African (green) populations**.** The frequency of allels in Estonian (blue) and UK (red) biobanks are shown in respectively colored dots.

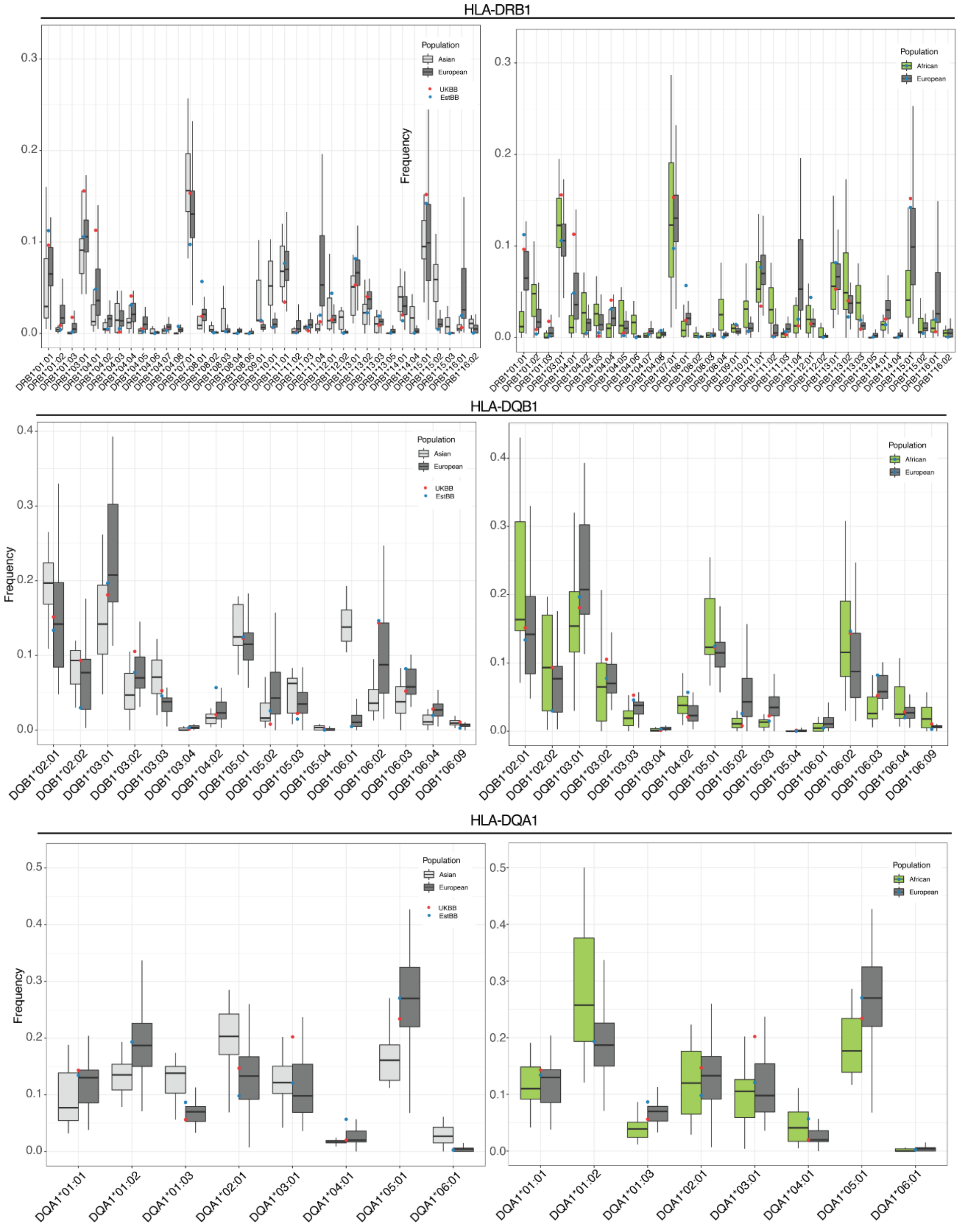

Figure S3. The distribution of allele frequencies of MHC class II genes HLA-DRB1, HLA-DQB1 and HLA-DQA1 in European (darker grey), Asian (lighter grey) and African (green) populations**.** The frequency of allels in Estonian (blue) and UK (red) biobanks are shown in respectively colored dots.

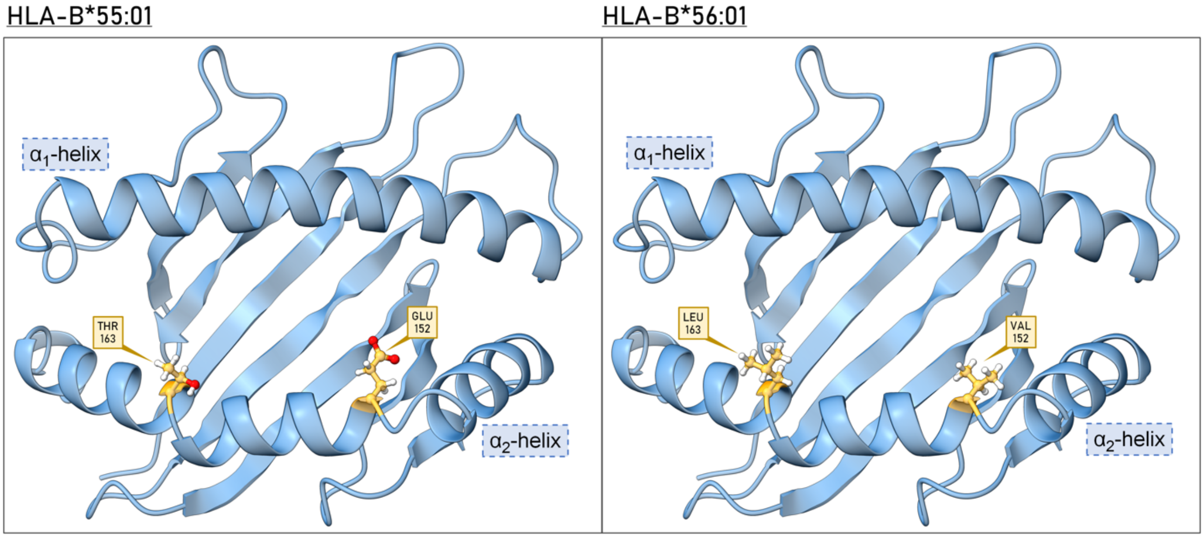

Figure S4. HLA-B*55:01 exhibits structural differences from another common HLA-B allele. Yellow residues highlight the two different amino acids in the antigen-binding cleft between 55:01 (left) and 56:01 (right).

Supplemental Tables

Table S1. Genome-wide significant associations of meta-analysis of penicillin allergy

Table S2. Associations of expression quantitative trait locus (eQTL) in blood with the results of penicillin allergy meta-analysis based on the eQTLGen Consortium data. eQTLGen is a meta-analysis of cis-/trans-eQTLs from 37 datasets with a total of 31,684 individuals. Signed stats column indicates the direction that is either "+" indicating that risk increasing allele increases the expression of the gene or "-" indicating the risk increasing allele decreases the expression of the gene.

Allergy/adverse effect of penicillin in PanUKB database	
**Chromosome**	6
**Position**	31369278
**rsID**	rs114892859
**Ref allele**	G
**Alt allele**	T
**European ancestry**	
**N cases**	20,021
**N Controls**	383,239
**Allele frequency of cases**	0.027
**Allele frequency of controls**	0.018
**Beta**	0.486
**Standard error**	0.041
**P-value**	$6,16x10^{-33}$
**African ancestry**	
**N cases**	298
**N Controls**	6,089
**Allele frequency of cases**	0.000
**Allele frequency of controls**	0.002
**Beta**	-1.081
**Standard error**	1.041
**P-value**	0.299
**Central/South Asian ancestry**	
**N cases**	434
**N Controls**	8,150
**Allele frequency of cases**	0.020
**Allele frequency of controls**	0.022
**Beta**	-0.114
**Standard error**	0.239
**P-value**	0.633
**East Asian ancestry**	
**N cases**	93
**N Controls**	2,535
**Allele frequency of cases**	NA
**Allele frequency of controls**	NA
**Beta**	NA
**Standard error**	NA
**P-value**	NA
**Middle Eastern ancestry**	
**N cases**	72
**N Controls**	1,478
**Allele frequency of cases**	0.035
**Allele frequency of controls**	0.021
**Beta**	0.311
**Standard error**	0.549
**P-value**	0.571

Table S3. Summary statistics of association of the rs114892859 variant with penicillin allergy in non-European ancestries based on the Pan-UKB database

Table S4. The frequencies of HLA four-digit alleles in Estonian and UK biobank.

Table S5. The frequency difference test between European vs Asian and European vs African populations using Wilcoxon test for all HLA alleles.

Table S6. Summary statistics of associations between penicillin allergy and four-digit HLA haplotypes in Estonian, UK and BioVU biobank.

Chromosome	Location	SNP	r_EstBB	r_UKBB
6	31369278	rs114892859	0.98	0.96
6	31374671	rs144626001	0.98	0.97
6	31352678	rs183120114	0.84	0.75
6	31371342	rs2301750	0.58	0.78
6	31326140	rs114492969	0.49	0.78
6	31325201	rs74194187	0.49	0.78
6	31326099	rs114734598	0.49	0.78
6	31326157	6_31326157	0.49	0.78
6	31326312	rs145887584	0.49	0.78
6	31326959	rs114166883	0.49	0.78
6	31327114	rs72502572	0.49	0.78
6	31327265	rs114783056	0.49	0.79
6	31327446	rs72502573	0.49	0.78
6	31327622	rs115200108	0.49	0.80
6	31336558	rs60177449	0.49	0.80
6	31342532	rs147336204	0.49	0.80
6	31344484	rs114654060	0.49	0.80
6	31344485	rs116355076	0.49	0.80
6	31348200	rs75931194	0.49	0.80
6	31334799	rs7766461	0.49	0.79
6	31321657	rs3177747	0.49	0.77
6	31321845	rs361531	0.49	0.77
6	31322767	rs3819284	0.49	0.77
6	31323414	rs41563818	0.49	0.70
6	31323707	rs41545339	0.49	0.71
6	31351321	rs72865324	0.49	0.80
6	31355813	rs2428484	0.49	0.80
6	31353285	rs72882965	0.49	0.80
6	31326178	rs184227609	0.48	0.70
6	31340628	rs72878037	0.48	0.80
6	31327326	rs68085422	0.48	0.79
6	31340795	rs72878039	0.48	0.79
6	31274734	rs114411923	0.34	0.28
6	31324764	rs41560522	0.30	0.41
6	31271783	rs145970926	0.29	0.27
6	31360838	rs142740116	0.29	0.27
6	31360289	rs72867732	0.29	0.27
6	31366969	rs72883110	0.28	0.27
6	31367052	rs146482045	0.28	0.27
6	31364848	rs72502580	0.28	0.29
6	31363312	rs140766555	0.28	0.22
6	31352694	rs56671953	0.27	0.26
6	31354939	rs72882972	0.27	0.26
6	31321184	rs72866766	0.22	0.25

Table S7. The HLA-B*5501 allele correlation with the SNPs in the HLA region in Estonian and UK biobank.

Table S8. Amino acid sequence similarity of 48 HLA-B allele serotypes present in Estonian and UK biobanks. Cells Green and yellow colouring indicates the similarity between values within columns (%), while blue and red colouring indicate a binary amino acid conservation within the specific column.

Trait	PMID	Category	Ethnicity	rg	se	z	p
Rheumatoid Arthritis	24390342	autoimmune	European	0.3531	0.0694	5.0866	3.65E-07
Asthma	17611496	autoimmune	European	0.3745	0.1235	3.0313	0.0024
Systemic lupus erythematosus	26502338	autoimmune	European	0.2182	0.1024	2.1321	0.0330
Multiple sclerosis	21833088	autoimmune	European	0.3558	0.1805	1.9713	0.0487
Primary biliary cirrhosis	26394269	autoimmune	European	0.1905	0.0971	1.9623	0.0497
Eczema	26482879	autoimmune	Mixed	0.2234	0.1219	1.8323	0.0669
Mean platelet volume	22139419	haemotological	European	0.1501	0.092	1.6311	0.1029
Heart rate	23583979	haemotological	Mixed	0.1117	0.0707	1.5804	0.1140
Inflammatory Bowel Disease (Euro)	26192919	autoimmune	European	0.079	0.0684	1.1552	0.2480
Crohns disease	26192919	autoimmune	European	0.0698	0.0712	0.9795	0.3273
Celiac disease	20190752	autoimmune	European	0.1049	0.1109	0.946	0.3441
Ulcerative colitis	26192919	autoimmune	European	0.0526	0.0777	0.6765	0.4987
Primary sclerosing cholangitis	27992413	autoimmune	Mixed	0.0104	0.0899	0.1157	0.9079
Platelet count	22139419	haemotological	European	-0.0084	0.0797	-0.1051	0,9163

Table S9. Genetic correlation of self-reported penicillin allergy with published autoimmune and hematological traits using LDHub. PMID – PubMed ID (reference study); rg- genetic correlation; se- standard error of rg; z- z-score of rg; p- p-value of rg.

Supplemental Methods

Phenotype definitions

All participants in both UK and Estonian Biobanks signed a consent form to allow follow-up linkage of their electronic health records (EHR), thereby enabling longitudinal collection of phenotypic information. EstBB allows access to the records of the national Health Insurance Fund Treatment Bills (since 2004), Tartu University Hospital (since 2008), and North Estonia Medical Center (since 2005). For every participant there is information on diagnoses in ICD-10 coding and drug dispensing data, including drug ATC codes, prescription status and purchase date (if available).

We extracted information on penicillin allergy by searching the records of the participants for Z88.0 ICD10 code. However, since the Z88.0 code seemed underreported in Estonia, we also used self-reported data on side-effects from penicillin for participants who reported hypersensitivity due to J01C* ATC drug group (Beta-Lactam Antibacterials, Penicillins) in their EstBB enrolment questionnaire. To validate this approach in EstBB we analyzed the effect of self-reported allergy status on the number on penicillin prescriptions in EstBB. We performed a Poisson regression among 37,825 unrelated individuals with J01C* prescriptions considering age, gender and 10 principal components (PC) as covariates. Units were interpreted as follows: 1-exp(beta)*100%=1-exp(-0.18)*100%= 16%. The Poisson model was considered appropriate as there was no large overdispersion.

To extract penicillin allergy from free-text we used a rule-based approach; the text had to contain any of the possible forms of the words ‘allergy’ or ‘allergic’ in Estonian as well as a potential variation of a penicillin name. As drug names are often misspelled, abbreviated or written using the English or Latin spelling instead of the standard

Estonian one, we used a regular expression to capture as many variations of each penicillin name as possible. In addition, we applied rules regarding the distance between the words 'allergy' and the drug name as well as other words nearby to exclude negations of penicillin allergies in the definition. This together with questionnaire data resulted in 1,320 cases with penicillin allergy.

For BioVU, penicillin allergies were extracted from the allergy sections of the clinical notes, which are often used to document a patient's intolerance or allergy to a drug as reported by the patient or observed by a healthcare provider.[1] The data in an allergy section in the clinical notes are semi-structured (e.g. penicillin [rash]). We defined penicillin allergy cases as individuals with any mention of the penicillin in the allergy section. Mentions of penicillin in the allergy section are identified using case-insensitive regular expressions that matched keywords for generic names, brand names, abbreviations (e.g., pcn), and common misspellings.

Genotype information

In brief, the 51,936 EstBB participants have been genotyped using the Global Screening Array v1 (GSA). Individuals were excluded from the analysis if their call-rate was < 95% or sex defined based on heterozygosity of X chromosome did not match sex in phenotype data. Variants were filtered by call-rate < 95% and HWE p-value < 1e-4 (autosomal variants only). Variant positions were updated to b37 and all variants were changed to be from TOP strand using tools and reference files provided in https://www.well.ox.ac.uk/~wrayner/strand/ webpage. Before imputation variants with MAF<1% and indels were removed. Phasing was done using Eagle v2.3

software[2] (number of conditioning haplotypes Eagle2 uses when phasing each sample was set to: --Kpbwt=20000) and imputation was done using Beagle v.28Sep18.793[3] using the Estonian population specific imputation reference panel constructed of 2,297 whole genome sequenced samples.

In UKBB genotype data are available for 488,377 participants of which 49,950 are genotyped using the Applied Biosystems™ UK BiLEVE Axiom™ and the remaining 438,427 individuals were genotyped using the Applied Biosystems™ UK Biobank Axiom™ Array by Affymetrix. The genotype data was phased using SHAPEIT3[4], and imputation was conducted using IMPUTE4[5] using a combined version of the Haplotype Reference Consortium (HRC) panel[6] and the UK10K panel.[7] We excluded individuals who have withdrawn their consent, have been labelled by UKBB to have poor heterozygosity or missingness, who have putative sex chromosome aneuploidy and who have >10 relatives in the dataset. We further removed all individuals with mismatching genetic and self-reported sex and ethnicity. GWAS was executed on individuals with confirmed white British ancestry.

Genotyping in Vanderbilt University Medical Center BioVU DNA Biobank was performed on the Infinium Multi-Ethnic Genotyping Array (MEGAchip). We excluded DNA samples: (1) with per-individual call rate < 95%; (2) with wrongly assigned sex; or (3) unexpected duplication. We performed whole genome imputation using the Michigan Imputation Server (https://imputationserver.sph.umich.edu)[8] with the Haplotype Reference Consortium, version r1.1[6], as reference. Principle components for ancestry (PCs) were calculated using common variants (MAF > 0.01) with high

variant call rate (> 98%), excluding variants in linkage and regions known to affect PCs (HLA region on chromosome 6, inversion on chromosome 8 (8135000-12000000) and inversion on chr 17 (40900000-45000000), GRCh37 build). For association analyses, we used EasyQC (www.genepi-regensburg.de/easyqc)[9] to filter (1) poorly imputed variants with imputation info r^2 value of < 0.5, (2) MAF < 0.005, (3) deviation from Hardy-Weinberg equilibrium with a P-value $\leq 1\times10^{-6}$ and (4) variants with MAF that deviated from the HRC reference panel by > 0.3.

Genome-wide study of penicillin allergy

Using the SAIGE software[10], we applied generalized mixed models with saddlepoint approximation to account for case-control imbalance and relatedness in all three cohorts. In EstBB the controls were selected from a set of individuals with no self-reported ADRs or with ICD10 diagnoses covered in a list of 79 ICD10 codes (described in [11]) with a possible drug-induced nature or diagnoses described as “due to drugs”. To minimize the effects of population admixture and stratification, the analyses only included samples with European ancestry based on PC analysis (PCA) and were adjusted for the first 10 PCs of the genotype matrix, as well as for birthyear and sex. In UKBB similarly as for EstBB, the GWAS was adjusted for the first 10 PCs of the genotype matrix, as well as for age and sex. In BioVU regression models were adjusted for sex, age, EHR length (years), and the first 10 principle components of the genotyping array for ancestry.

Post-GWAS annotation

FUMA (Functional mapping and annotation of genetic associations)[12] is an integrative web-based platform using information from multiple biological resources, including e.g. information on eQTLs, chromatin interaction mappings, and LD structure to annotate GWAS data. We applied FUMA to identify lead SNPs and genomic risk loci for results of the meta-analysis, using the European LD reference panel from 1000G.[13] Further eQTL associations were identified based on data from the eQTLGen consortium, which is a meta-analysis of 37 datasets with blood gene expression data pertaining to 31,684 individuals.[14]

HaploReg[15] was used for exploring annotations, chromatin states, conservation, and regulatory motif alterations. To estimate the relative deleteriousness of the identified SNPs, we used the Combined Annotation Dependent Depletion (CADD) framework.[16]

HLA typing

The SNP2HLA tool imputes HLA alleles from SNP genotype data and single Nucleotide Variants (SNVs), small INsertions and DELetions (INDELs) and classical HLA variants were called using whole genome sequences of 2,244 study participants from the Estonian Biobank sequenced at 26.1x. We performed high-resolution (G-group) HLA calling of three class-I HLA genes (HLA-A, -B and -C) and three class-II HLA genes (HLA-DRB1, -DQA1 and -DQB1) using the HLA*PRG algorithm.[17] SNVs and INDELs were called using GATK version 3.6 according to the best practices for variant discovery.[18] Classical HLA alleles, HLA amino acid residues and untyped SNPs were then imputed using SNP2HLA and the reference panel constructed using the 2,244 whole-genome sequenced Estonian samples. We performed an additive

logistic regression analysis with the called HLA alleles using R *glm* function in EstBB including age, sex and 10 PCs as covariates.

In UKBB, for each genotype call one metric is reported, the absolute posterior probability of the allele inference. We applied thresholding to the maximum posterior probability (at a threshold of 0.8) to create a marker representing the presence/absence of each HLA allele for each individual participant. Only alleles with a minor allele frequency of > 0.01% were included in the analysis, amounting to 202 alleles taken forward for association testing with penicillin allergy. In UKBB we performed association analysis of each four-digit allele with the Z88.0 subcode using logistic regression function *glm* in R, adjusting for sex, age, age^2, recruitment center, genotyping array, and the first 15 principal components (and excluding one individual of each pair of related [up to 2nd degree or closer, using KING's kinship coefficient > 0.0884] individuals and those of reported non-white ancestry).

For BioVU, SNP2HLA was used to impute four-digit HLA A B C DP DR DQ typing from SNP data from the MEGAchip. We performed an additive logistic regression analysis with the called HLA alleles using R *glm* function in BioVU including age, sex, EHR length (years), and 10 PCs as covariates.

Comparison of HLA allele frequencies

To compare obtained frequencies of HLA alleles with reported frequencies in European, Asian and African populations we used the database of Allele Frequencies of worldwide populations (http://www.allelefrequencies.net/default.asp). We queried

the frequencies of four-digit alleles choosing the following regions: Europe, North-East Asia, South-Asia, South-East Asia, Western Asia, North Africa and Sub-Saharan Africa. Frequency comparisons were visualized with R software (3.3.2)[19] using ggplot2 package and frequency difference was calculated with two-samples Wilcoxon test.

Replication in 23andMe

All individuals included in the analyses provided informed consent and participated in the research online, under a protocol approved by the external AAHRPP-accredited IRB, Ethical & Independent Review Services (E&I Review). Penicillin allergy was determined based on the survey questions for allergic symptoms or for questions related to allergy test. Survey questions for positive allergy test were "Have you ever had a positive allergy test to any of these medications? [CONCEPT: penicillin]"; or "Has a doctor confirmed that you had an allergic reaction to penicillin, amoxicillin, or ampicillin?". A logistic regression assuming an additive model for allelic effects was used with adjusting for age, sex, indicator variables to represent the genotyping platforms and the first five genotype principal components. In the 23andMe replication study, the HLA imputation was performed by using HIBAG [20] with the default settings. We imputed allelic dosage for HLA-A, B, C, DPB1, DQA1, DQB1 and DRB1 loci at four-digit resolution[21].

HLA-B*55:01 allele association with lymphocyte levels in EstBB

To study the association between the HLA-B*55:01 allele and lymphocyte levels in EstBB, we extracted the information on measured lymphocyte levels (number of cells per nanoliter) from the free text fields of the medical history of 4,567 unrelated

individuals with genotype data. After removing outliers based on the values of any data points which lie beyond the extremes of the whiskers (values > 3.58 and < 0.26), a linear regression was performed using R software and with age and sex as covariates.

Comparison of the amino acid sequences of HLA-B alleles

The sequences for the common 48 HLA-B variants within EstBB and UKBB were acquired from the IPD-IMGT/HLA database,[22] which subsequently were aligned with NCBI Protein BLAST.[23] The molecular structures for HLA-B*55:01 and HLA-B*56:01 were created via SWISS-MODEL[24] and visualized with UCSF ChimeraX[25], developed by the Resource for Biocomputing, Visualization, and Informatics at the University of California, San Francisco, with support from National Institutes of Health R01-GM129325 and the Office of Cyber Infrastructure and Computational Biology, National Institute of Allergy and Infectious Diseases.

Supplemental Acknowledgements

Computation used the Oxford Biomedical Research Computing (BMRC) facility, a joint development between the Wellcome Centre for Human Genetics and the Big Data Institute supported by Health Data Research UK and the NIHR Oxford Biomedical Research Centre. Financial support was provided by the Wellcome Trust Core Award Grant Number 203141/Z/16/Z. The views expressed are those of the authors and not necessarily those of the NHS, the NIHR or the Department of Health.

Supplemental References

1. Zhou, L., Dhopeshwarkar, N., Blumenthal, K.G., Goss, F., Topaz, M., Slight, S.P., and Bates, D.W. (2016). Drug allergies documented in electronic health records of a large healthcare system. Allergy Eur. J. Allergy Clin. Immunol. *71*, 1305–1313.

2. Loh, P.R., Danecek, P., Palamara, P.F., Fuchsberger, C., Reshef, Y.A., Finucane, H.K., Schoenherr, S., Forer, L., McCarthy, S., Abecasis, G.R., et al. (2016). Reference-based phasing using the Haplotype Reference Consortium panel. Nat. Genet. *48*, 1443–1448.

3. Browning, S.R., and Browning, B.L. (2007). Rapid and accurate haplotype phasing and missing-data inference for whole-genome association studies by use of localized haplotype clustering. Am. J. Hum. Genet. *81*, 1084–1097.

4. O'Connell, J., Sharp, K., Shrine, N., Wain, L., Hall, I., Tobin, M., Zagury, J.F., Delaneau, O., and Marchini, J. (2016). Haplotype estimation for biobank-scale data sets. Nat. Genet. *48*, 817–820.

5. Bycroft, C., Freeman, C., Petkova, D., Band, G., Elliott, L.T., Sharp, K., Motyer, A., Vukcevic, D., Delaneau, O., O'Connell, J., et al. (2018). The UK Biobank resource with deep phenotyping and genomic data. Nature *562*, 203–209.

6. McCarthy, S., Das, S., Kretzschmar, W., Delaneau, O., Wood, A.R., Teumer, A., Kang, H.M., Fuchsberger, C., Danecek, P., Sharp, K., et al. (2016). A reference panel of 64,976 haplotypes for genotype imputation. Nat. Genet. *48*, 1279–1283.

7. Walter, K., Min, J.L., Huang, J., Crooks, L., Memari, Y., McCarthy, S., Perry, J.R.B., Xu, C., Futema, M., Lawson, D., et al. (2015). The UK10K project identifies rare variants in health and disease. Nature *526*, 82–89.

8. Das, S., Forer, L., Schönherr, S., Sidore, C., Locke, A.E., Kwong, A., Vrieze, S.I.,

Chew, E.Y., Levy, S., McGue, M., et al. (2016). Next-generation genotype imputation service and methods. Nat. Genet. *48*, 1284–1287.

9. Winkler, T.W., Day, F.R., Croteau-Chonka, D.C., Wood, A.R., Locke, A.E., Mägi, R., Ferreira, T., Fall, T., Graff, M., Justice, A.E., et al. (2014). Quality control and conduct of genome-wide association meta-analyses. Nat. Protoc. *9*, 1192–1212.

10. Zhou, W., Nielsen, J.B., Fritsche, L.G., Dey, R., Gabrielsen, M.E., Wolford, B.N., LeFaive, J., VandeHaar, P., Gagliano, S.A., Gifford, A., et al. (2018). Efficiently controlling for case-control imbalance and sample relatedness in large-scale genetic association studies. Nat. Genet. *50*, 1335–1341.

11. Tasa, T., Krebs, K., Kals, M., Mägi, R., Lauschke, V.M., Haller, T., Puurand, T., Remm, M., Esko, T., Metspalu, A., et al. (2019). Genetic variation in the Estonian population: pharmacogenomics study of adverse drug effects using electronic health records. Eur. J. Hum. Genet. *27*, 442–454.

12. Watanabe, K., Taskesen, E., Van Bochoven, A., and Posthuma, D. (2017). Functional mapping and annotation of genetic associations with FUMA. Nat. Commun. *8*, 1–11.

13. Auton, A., Abecasis, G.R., Altshuler, D.M., Durbin, R.M., Bentley, D.R., Chakravarti, A., Clark, A.G., Donnelly, P., Eichler, E.E., Flicek, P., et al. (2015). A global reference for human genetic variation. Nature *526*, 68–74.

14. Võsa, U., Claringbould, A., Westra, H.-J., Bonder, M.J., Deelen, P., Zeng, B., Kirsten, H., Saha, A., Kreuzhuber, R., Kasela, S., et al. (2018). Unraveling the polygenic architecture of complex traits using blood eQTL metaanalysis. BioRxiv 447367.

15. Ward, L.D., and Kellis, M. (2012). HaploReg: a resource for exploring chromatin states, conservation, and regulatory motif alterations within sets of genetically linked

variants. Nucleic Acids Res. *40*, D930–D934.

16. Rentzsch, P., Witten, D., Cooper, G.M., Shendure, J., and Kircher, M. (2019). CADD: predicting the deleteriousness of variants throughout the human genome. Nucleic Acids Res. *47*, D886–D894.

17. Dilthey, A.T., Gourraud, P.-A., Mentzer, A.J., Cereb, N., Iqbal, Z., and McVean, G. (2016). High-Accuracy HLA Type Inference from Whole-Genome Sequencing Data Using Population Reference Graphs. PLOS Comput. Biol. *12*, e1005151.

18. Broad Institute GATK | Germline short variant discovery (SNPs + Indels).

19. R Core Team (2018). R: a language and environment for statistical computing.

20. Zheng, X., Shen, J., Cox, C., Wakefield, J.C., Ehm, M.G., Nelson, M.R., and Weir, B.S. (2014). HIBAG - HLA genotype imputation with attribute bagging. Pharmacogenomics J. *14*, 192–200.

21. Tian, C., Hromatka, B.S., Kiefer, A.K., Eriksson, N., Noble, S.M., Tung, J.Y., and Hinds, D.A. (2017). Genome-wide association and HLA region fine-mapping studies identify susceptibility loci for multiple common infections. Nat. Commun. *8*, 1–13.

22. Robinson, J., Barker, D.J., Georgiou, X., Cooper, M.A., Flicek, P., and Marsh, S.G.E. (2020). IPD-IMGT/HLA Database. Nucleic Acids Res. *48*, D948–D955.

23. Protein BLAST: search protein databases using a protein query.

24. Grosdidier, A., Zoete, V., and Michielin, O. (2011). SwissDock, a protein-small molecule docking web service based on EADock DSS. Nucleic Acids Res. *39*, W270-7.

25. Goddard, T.D., Huang, C.C., Meng, E.C., Pettersen, E.F., Couch, G.S., Morris, J.H., and Ferrin, T.E. (2018). UCSF ChimeraX: Meeting modern challenges in visualization and analysis. Protein Sci. *27*, 14–25.
